# Modeling Rumor Diffusion Process With the Consideration of Individual Heterogeneity: Take the Imported Food Safety Issue as an Example During the COVID-19 Pandemic

**DOI:** 10.3389/fpubh.2022.781691

**Published:** 2022-03-07

**Authors:** Tinggui Chen, Jingtao Rong, Jianjun Yang, Guodong Cong

**Affiliations:** ^1^School of Statistics and Mathematics, Zhejiang Gongshang University, Hangzhou, China; ^2^Academy of Zhejiang Culture Industry Innovation & Development, Hangzhou, China; ^3^Department of Computer Science and Information Systems, University of North Georgia, Oakwood, GA, United States; ^4^School of Tourism and Urban-Rural Planning, Zhejiang Gongshang University, Hangzhou, China

**Keywords:** individual heterogeneity, rumor diffusion, SEIR-OM model, rumor control, COVID-19 pandemic

## Abstract

At present, rumors appear frequently in social platforms. The rumor diffusion will cause a great impact on the network order and the stability of the society. So it's necessary to study the diffusion process and develop the rumor control strategies. This article integrates three heterogeneous factors into the SEIR model and designs an individual state transition mode at first. Secondly, based on the influencing factors such as the trust degree among individuals, an individual information interaction mode is constructed. Finally, an improved SEIR model named SEIR-OM model is established, and the diffusion process of rumors are simulated and analyzed. The results show that: (1) when the average value of the interest correlation is greater, the information content deviation is lower, but the rumor diffusion range will be wider. (2) The increase of the average network degree intensifies influence of rumors, but its impact on the diffusion has a peak. (3) Adopting strategies in advance can effectively reduce the influence of rumors. In addition, the government should enforce rumor-refuting strategies right after the event. Also, the number of rumor-refuting individuals must be paid attention to. Finally, the article verifies the rationality and effectiveness of the SEIR-OM model through the real case.

## Introduction

With the rapid development of Internet information technology, information diffusion has become more and more convenient. However, due to malicious tampering and other reasons, information will continue to be alienated in the diffusion process, which will increase its complexity and redundancy. While receiving a large amount of information, netizens cannot verify its authenticity and accuracy. This provides an opportunity for the large-scale rumor diffusion. At this time, if the hot information related to the national economy and people's livelihood is tampered with and not controlled in time, it will easily breed public anxiety, panic and other emotions, which will bring great economic impact to individuals, society or the country, and even threaten the harmony and stability of society. For example, when COVID-19 broke out at the end of 2019, rumors that “masks cannot prevent viruses” diffuse on social platforms in many countries, thus many people failed to take correct epidemic prevention measures in time, causing the widespread diffusion of COVID-19 in many countries, which greatly affected the social and economic development in the world. As a result, analyzing the rumor formation, diffusion and its control strategy has an important theoretical and practical significance.

Scholars have conducted a lot of research on the diffusion and control of rumors and have achieved some results. At present, the research on rumors is mainly divided into two categories: (1) A qualitative analysis of the diffusion process of rumors from the phenomenon itself, mainly to study its causes and counter measures. However, most of these studies lack specific empirical investigations and quantitative methods, and their conclusions are subjective; (2) Use evolutionary game theory, communication dynamics and other related methods to construct mathematical models, and use mathematical derivation or computer simulation to achieve inter-group interactive simulation of information diffusion, and observe the results to explore the rules of rumor diffusion and counter measures.

However, most of these models simulate the diffusion process of rumors, but rarely consider the formation of rumors and psychological influence factors. Based on this, this article integrates the individual's diffusion willingness, the individual's forgetting degree, and the intensity of government punishment into the SEIR model, and designs a state transition mode at first. Secondly, it considers the individual's decision-making behavior in the process of rumor generation and diffusion, and establishes information interaction mode among individuals. Finally, an improved SEIR model named SEIR-OM model is established. Also, rumor generation and diffusion process are simulated and analyzed from two aspects: model parameter setting and rumor control strategy.

The structure of the article is organized as follows: section Literature Review is a literature review. Section Research Framework builds a SEIR-OM model. Section Model Construction simulates the rumor evolution process through simulation experiments, and studies the influences of model parameters and different rumor control strategies on the rumor evolution. Section Simulation Experiment validates the SEIR-OM model with the real case from the imported food safety issue during the COVID-19 Pandemic. Section Empirical Analysis makes the conclusions and prospects for future work.

## Literature Review

This section reviews the relevant literature from two aspects: rumor diffusion and control.

With regard to the research on the rumor diffusion, most of the existing literature uses infectious disease models and evolutionary game models to analyze their diffusing process: (1) the first aspect is the research on the dynamics of rumor diffusion based on the infectious disease model. For instance, Zhang and Zhu (1) studied two kinds of rumor diffusing dynamics with quadratic relationship by establishing the I2S2R model, and concluded that the diffusing intensity of second rumors depended on the diffusing intensity of initial rumors. In addition, based on the SIR model, Huang and Jin (2) divided the immunized population into two categories: those who accepted rumors but were not interested in diffusing them, and those who did not believe rumors, and analyzed two strategies through numerical simulation: random immunization and target immunization. The results showed that the application of random or directed immunity could effectively prevent the diffusion of rumors while reducing the credibility of rumors. Jiang and Yan (3) proposed a piecewise SIR model to quantify the diffusing speed, scale and influence of online information. The simulation results showed that there was no proportional relationship between the sustained influence of a message and the number of diffusers. Zhou et al. (4) analyzed the influence of network topology on rumor diffusion based on SIR model. The mean field analysis showed that the number of infected nodes depended on the network topology. Moreno et al. (5) studied the dynamic process of rumor evolution in homogeneous network and scale-free network. The results showed that when rumor diffused in the latter, the number of people who did not diffuse rumor in the final state had nothing to do with the degree of the source of infection, but was closely related to the probability of infection. Zhang et al. (6) considered the influence of the attractiveness of information itself on the diffusion, and based on this, they proposed a rumor diffusion model based on the diffusion ability. Most of the above-mentioned literatures have added more diverse individual states on the basis of classic infectious disease models. However, since the individual interaction mechanism in the process of rumor diffusion is not considered, most studies still use fixed reception probability to describe the process of individuals receiving external information. (2) The second aspect is to use the evolutionary game model to describe the game decision-making process of individuals facing rumors. For instance, Fernández-Domingos et al. (7) established a prisoner's dilemma game model, and analyzed the behavior of each node in the topology during network information diffusion. This study showed that in small-scale networks, choosing cooperation was the optimal strategy of nodes. On the contrary, for large-scale networks, choosing non-cooperation was the optimal strategy. Furthermore, by using three real social network datasets, Li et al. (8) found that increasing the judgment ability of individuals could curb the diffusion of rumor effectively. Moreover, there existed some optimal risk coefficients and punishment fractions that could help more people refuse to diffuse rumor. Mojgan et al. (9) proposed an evolutionary game model to analyze the diffusion process of rumors in social networks. The model studied the factors affecting people's decision-making, such as social anxiety, and conducted sensitivity analysis experiments to illustrate the impact of different factors on the process of rumor propagation. The analysis showed that people's attitude toward rumor/anti-rumor had a significant impact on rumor diffusion. In addition, factors such as social anxiety and rumor intensity also accelerated the rumor diffusion. Most of the above-mentioned documents have studied the diffusion process of rumors among individuals on the Internet, but rarely studied the process of their formation, which cannot fully reflect the large-scale diffusion process of rumors from its initiation, and from weak to strong of the whole evolution. However, the research on the formation mechanism of rumors can effectively reduce the generation of rumors, which is very important for rumor control. Therefore, it is necessary to study the formation mechanism of rumors.

In addition, regarding the research on rumor control, the methods used in the existing literature mainly include controlling high-influence nodes, controlling key connecting edges, and diffusing refuting information. The details are as follows: (1) Control high-influence nodes. This type of method aims to find nodes that contribute to the rumor diffusion, and then delete such nodes to reduce the influence of rumors. Some typical literature is as follows: based on a variety of complex network metrics of network centrality, e.g., centrality of degree, intermediate, proximity, etc., Comin et al. (10) analyzed three communication mechanisms and provided an effective method of hairstyle communication sources. Inspired by the idea of gravity formula, Ma et al. (11) took the *k*-shell value of the node as its mass and the shortest path length between the two nodes as the distance, proposed the gravity centrality method to determine the high influence node, and compared it with other centrality indexes. (2) Control key connecting edges. This type of method aims to find the edges that play key nodes in information dissemination and delete them to reduce the rumor diffusion. Some typical literature is as follows: Pallis (12) deleted *k* edges from the original network to diffuse rumors as little as possible, and explained which edge should be deleted depended on the eigenvalues of the network adjacency matrix. Yuan et al. (13) proposed a fine-grained heuristic algorithm to solve the rumor propagation minimization problem. The experiment showed that the heuristics based on betweenness and out-degree were orders of magnitude faster than the greedy algorithm in terms of running time. (3) Diffusing refuting information. This type of method diffuses information that is contrary to the content of the rumors, so that as many nodes as possible are not deceived by the rumors. Some typical literature is as follows: Zhang et al. (14) presented an in-depth analysis of the function of official rumor-refuting information (ORI) in suppressing and quashing rumors. They determined the influencing factors and constructed a competition model. The simulation results also indicated that government credibility and the release time of ORI played a critical role in controlling rumors. Zhang and Xu (15) presented a simple model to describe the interplay between rumors and rumor-refuting information based on biomathematics theory. By drawing from differential equations, a theoretical analysis reveals that this model exhibited three dynamic cases: extinction of rumors, extinction of rumor-refuting information and coexistence. Also, they studied the stability of the equilibrium points of three cases, found that stable condition of equilibrium point, and showed unstable case of model. Most of the above-mentioned literature studies the effects of different rumor control strategies adopted after the occurrence of hot events, but few literature explores the role of rumor prevention strategies adopted before the occurrence. However, proactive prevention strategy is also an important part of rumor control strategy, so it is necessary to study it.

To sum up, the academies have conducted a certain depth of research on the diffusion and control of rumors, but there are still deficiencies. Based on this, in section Model Construction, this article first designs a state transition mode based on SEIR model. At the same time, considering the rumor generating factors such as information tampering and individual heterogeneity factors such as personal reputation, an information interaction mode is constructed. Finally, SEIR-OM model is constructed by fusing state transition mode and information interaction mode. In addition, this article also divides the rumor control strategy into proactive strategy and reactive rumor refutation strategy, and analyzes their effects through simulation experiments.

## Research Framework

**Figure 1:**
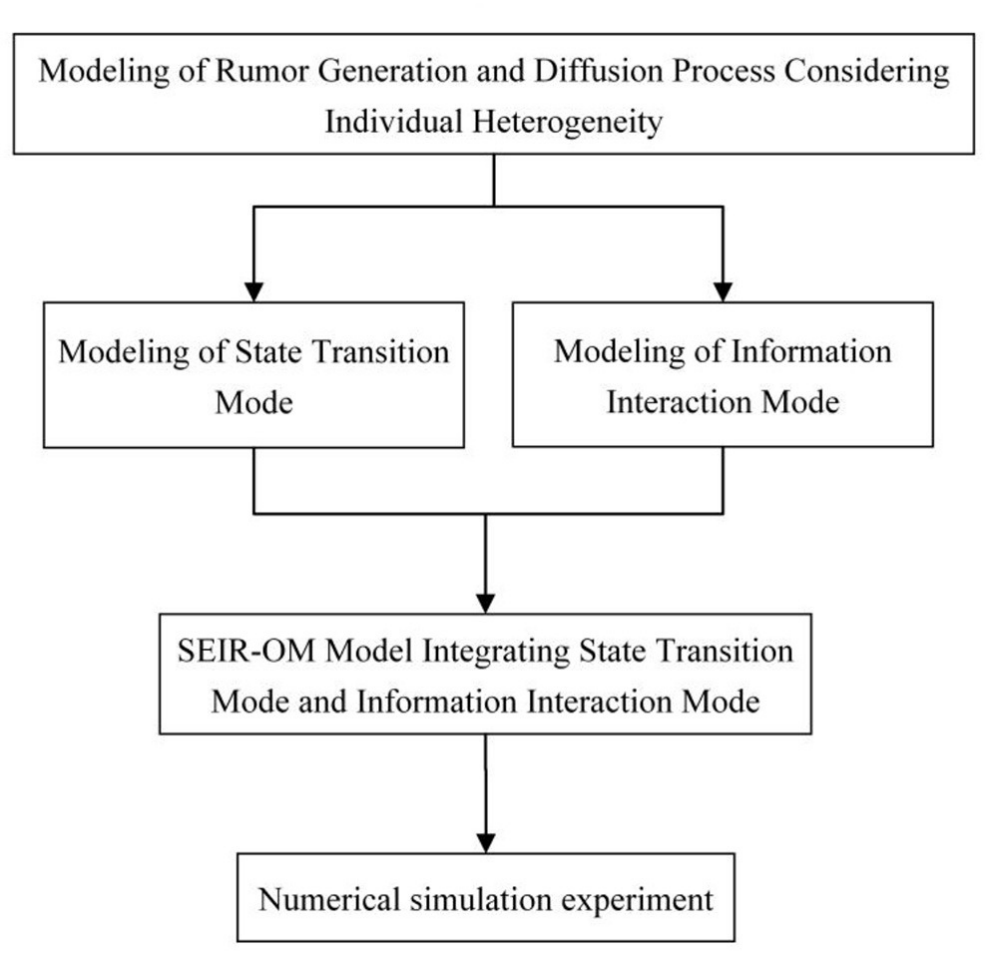
Framework of this article.

This article integrates the individual's diffusion willingness, the individual's forgetting degree, and the intensity of government punishment into the SEIR model, and designs the state transition mode at first. Secondly, it refers to the trust theory and information asymmetry theory, considers the main factors affecting information interaction among individuals, and establishes information interaction mode. Finally, an improved SEIR model named SEIR-OM model is constructed, and its formation and diffusing process are simulated and analyzed from two aspects: model parameter setting and rumor control strategy. The framework of the article is shown in Figure 1.

## Model Construction

### Classic SEIR Model

SEIR model is a classical infectious disease dynamics model, which is often used in the medical field to simulate the transmission process of infectious diseases (16, 17) and predict the development trend of epidemic situation (18, 19). The dissemination of public opinion information needs go through the process of germination, development, outbreak and finally decline, which is highly similar to the whole process of the development of infectious diseases. Therefore, in the existing research on information dissemination, a considerable proportion of studies uses SEIR model to analyze information dissemination.

The classic SEIR model divides individuals into four categories according to their different states in the diffusion process, namely: uninformed individual S, silent individual E, communication individual I, and immune individual R. Among them, uninformed individuals represent those who have not received information, corresponding to those who do not know the public opinion information in reality, and the initial states of most individuals are uninformed states; silent individuals represent those who have received information but have not diffused to uninformed ones; communication individuals represent those who receive information and diffuse information to other ones; immune individuals represent those referring to individuals who are no longer interested in information related to the event, which are the final states of individuals.

Moreover, the classic SEIR model has four assumptions: (1) The number of individuals always remains a constant, i.e., S+E+I+R=N (N is a constant); (2) Uninformed individuals turn into the silent after receiving information from the communication individual. Therefore, at *t* + 1, the number of newly-added silent ones is proportional to the number of communication ones at time *t*, and its proportional coefficient α is defined as the reception coefficient; (3) The number of newly-added silent ones at *t* + 1 is proportional to the total number of silent ones at time *t*, and its proportional coefficient σ is defined as the diffusing coefficient; (4) The communication individuals turn into immune ones after losing interest in the event-related information. Therefore, at time *t* + 1, the number of newly immunized individuals is proportional to the number of communication ones at time *t*, and the proportional coefficient ρ is defined as the immune coefficient. Based on the above four assumptions, the differential equations of the SEIR model are shown in formula (1):


(1) $$\begin{array}{l} \{\begin{array}{c} \frac{d\text{S}\left(t\right)}{dt}=-\alpha \text{I}\left(t\right)\text{S}\left(t\right)\\ \frac{d\text{E}\left(t\right)}{dt}=\alpha \text{I}\left(t\right)\text{S}\left(t\right)-\sigma \text{E}\left(t\right)\\ \frac{d\text{I}\left(t\right)}{dt}=\sigma \text{E}\left(t\right)-\rho \text{I}\left(t\right)\\ \frac{d\text{R}\left(t\right)}{dt}=\rho \text{I}\left(t\right) \end{array} \end{array}$$

**Figure 2:**
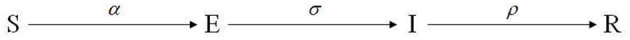
Classic SEIR model.

Figure 2 is a schematic diagram of the classic SEIR model:

SEIR model uses fixed probability to describe the individual state transition process and information interaction process in the process of rumor propagation, ignoring the influence of individual heterogeneity factors on the process of rumor propagation. Based on this, next section will improve the classic SEIR model and construct the SEIR-OM model.

### SEIR-OM Model Construction

In this section, the construction process of SEIR-OM model will be described in detail. The parameters and variables involved in the model are shown in Tables 1, 2.

**Table 1:** Involved parameters in the model.

**Parameter**	**Description**	**Value**
*w*	Diffusion threshold (refers to the critical value of information diffusion to the outside world)	[0,1]
*f*	Forgetting threshold (refers to the critical value of forgetting events)	[1,+∞)
*b_*it*_*	Degree of interest correlation between individual *i* and public opinion events at time *t*	[0,1]
*μ_*b*_*	Mean value of the degree of interest correlation between all individuals and public opinion events	[0,1]
*s_*b*_*	Standard deviation of interest correlation between all individuals and public opinion events	[0,+∞)
*c_*i*_*	Trust threshold of individual *i* (refers to the threshold at which the individual chooses to trust other individuals)	[0,1]
*μ_*c*_*	Mean value of the trust thresholds of all individuals	[0,1]
*p*	Government punishment on rumors	[0,1]
*m_*it*_*	Accumulated gain due to external feedback after the information diffusion	[0,+∞]
*v_*it*_*	Amount of information received by individual *i* at time *t*	[0,+∞]
*N*	Total number of individuals in the network	(0,+∞)
*s_*ij*_*	Shortest path between individual *i* and *j*	[0,+∞]
*k_*i*_*	Number of neighbors of individual *i*	[0,+∞]
*n_*ij*_*	Number of common neighbors of individuals *i* and *j*	[0,+∞]
*d*_1*i*_	Subject deviation of the information content between mastered by individual *i* and original information	[0,2]
*d*_2*i*_	Predicate deviation of the information content between mastered by individual *i* and original information	[0,2]
*d*_3*i*_	Object deviation of the information content between mastered by individual *i* and original information	[0,2]
*d*_4*i*_	Attribute deviation of the information content between mastered by individual *i* and original information	[0,2]
*d*_5*i*_	Adverbial deviation of the information content between mastered by individual *i* and original information	[0,2]
*s*_1*i*_	Deviation between the subject of the information content transmitted by individual *i* and that of the original information content	[0,2]
*s*_2*i*_	Deviation between the predicate of the information content transmitted by individual *i* and that of the original information content	[0,2]
*s*_3*i*_	Deviation between the object of the information content transmitted by individual *i* and that of the original information content	[0,2]
*s_4*i*_*	Deviation between the attribute of the information content transmitted by individual *i* and that of the original information content	[0,2]
*s*_5*i*_	Deviation between the adverbial of the information content transmitted by individual *i* and that of the original information content	[0,2]

**Table 2:** Involved variables in the model.

**Variable**	**Description**	**Value**
*W_*i*_*	Individual *i*'s diffusion willingness	[0,1]
*F_*i*_*	Individual *i*'s forgetting degree	[0,1]
*D_*i*_*	The set of deviations between the information content mastered by individual *i* and the original information content	
*S_*i*_*	The set of deviations between the information content diffused by individual *i* and the original information content	
*I_*i*_*	Social influence of individual *i*	[0,1]
*R_*ij*_*	Strength of the relationship between individuals *i* and *j*	[0,1]
*C_*ij*_*	The trust degree of individual *i* to individual *j*	[0,1]
*K_*i*_*	Knowledge reserve of individual *i*	[0,1]
Σ_*i*_	The degree of confusion of external information received by individual *i* in past information interactions	[0,1]
*G_*i*_*	Individual *i*'s mastery of event-related information	[0,1]
Δ_*i*_	Individual *i*'s tampered intensity with information content	[0,1]

#### State Transition Mode

The SEIR model uses a fixed probability to describe the transition of an individual's state, without considering the heterogeneity of the individual, so it cannot explain the internal mechanism of the individual's state transition. Based on this, the individual state transition mode of the SEIR model is improved here, and two factors describing individual heterogeneity are introduced, namely: individual's diffusion willingness and the individual's forgetting degree, which are used as the conditions for individual state transition.

(1) Individual's diffusion willingness. Diffusion willingness refers to “the sending intensity of sender's personal consciousness” (20), which is used to determine whether the individual diffuses the information to the outside world. It is important to determine whether the information can be diffused on a large scale in social networks. Generally, the factors that affect the one's diffusion willingness include two aspects: one is the degree of interest correlation between the individual and the event, which refers to the degree of influence of the occurrence and development of an event on a certain aspect of the person's interests (21), e.g., the occurrence of public health emergencies will damage the personal interests of local residents. The higher interest correlation of the individual to the event indicates the stronger willingness to diffuse relevant information; the other is accumulated gains due to external feedback after the information diffusion. If other individuals receive the information diffused by this individual, this individual's diffusion gains will increase, and his willingness to diffuse the information will be stronger. However, if other individuals reject the information diffused by the individual, his/her diffusion gains will decrease, and the corresponding diffusion willingness is also weaker. Therefore, the individual's diffusion willingness *W*_*i*_ is described by formula (2) (22):


(2) $$\begin{array}{l} W_{i}\left(t\right)=\left(b_{it}-1\right)e^{1-m_{it}}+\left(1-p\right) \end{array}$$

where *b*_*it*_ = *b*_*i*(t−1)_ + 0.1*v*_*it*_. Because each individual has a difference in the degree of interest correlation to a certain event, we assume that *b*_*i*0_ obeys a normal distribution with a mean value of μ_*b*_ and a variance of *b*^2^, and is mapped to the interval [0,1]. *m*_*i*0_ = 1, when other individuals receive the information sent by individual *i*, and *m*_*i*_ is increased by 1.

(2) Individual's forgetting degree. Individual's attention to hot events will decay over time. Ebbinghaus research found that the failing of people's memory is fast at first and then slower. Considering that the degree of interest between individuals and the event will affect their attention to the event, referring to the Ebbinghaus forgetting curve equation, the individual forgetting degree *F*_*i*_ is described by the formula (3):


(3) $$\begin{array}{l} F_{i}\left(t\right)=1-e^{-\frac{t}{b_{it}}} \end{array}$$

Similar to the classical SEIR model, SEIR-OM model also divides individuals into four categories: uninformed individuals S, silent individuals E, communication individuals I, and immune individuals R. They also have the same meaning as the classical SEIR model.

**Figure 3:**
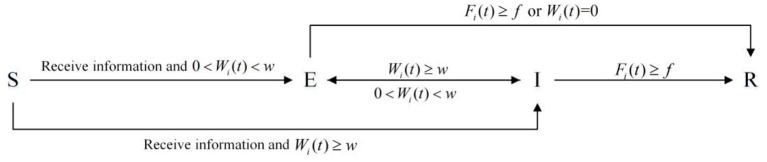
Individual state transition rule.

In the individual state transition mode, the state transition rules are set as follows: when an uninformed person interacts with a communication one, the uninformed individual will transform into a silent one or a communication one according to his diffusion willingness. When the silent individual's diffusion willingness is greater than or equal to the diffusion threshold *w*, it turns into a communication one. When a communication individual's willingness is less than the diffusing threshold *w* and >0, he/she turns into a silent individual. If the individual's diffusion willingness is <0 or the forgetting degree is greater than forgetting threshold *f*, he/she turns into an immune one. The individual state transition rule is shown in Figure 3.

Note that although uninformed individuals and immune individuals do not participate in information dissemination, there are some differences between them. The uninformed individual means that the initial state of most individuals is uninformed state. After receiving the information, the state of the uninformed individual will change. On the contrary, the silent individual means that the final state of most individuals is silent state, and it will not change again. Also, the silent individuals will disconnect from other individuals.

#### Information Interaction Mode

The large-scale rumor diffusion is inseparable from the information interaction among individuals, and the information interaction process includes two stages, namely: the receiving stage and the diffusion stage of information. Existing studies mostly use SEIR model and evolutionary game model to describe this process. However, the SEIR model describes this process through fixed reception probabilities and diffusion probabilities, ignoring the influence of individual heterogeneity factors on the information interaction process. While in the evolutionary game model, individuals choose whether to receive and diffuse information only based on the diffusion benefits. In addition, both the SEIR model and the evolutionary game model only describe the diffusion process after rumors are generated, and do not consider the rumor generation mechanism. Based on this, an information interaction mode is designed here to reflect the process of rumor generation and information interaction.

##### Information Content Deviation

Different people have different positions and opinions on the same public opinion event, and there are situations in the network where individuals distort and fabricate real information to gain attention. Therefore, in the process of information diffusion, information deviation is often caused, and a variety of different content of information coexist. In order to differentiate the information content mastered by different people and describe the difference between them and the original information content, the information content deviation set is established according to the Chinese sentence structure here.

In the Chinese context, a sentence is mainly composed of five parts, namely: subject, predicate, object, attributive, and adverbial. Therefore, the information content deviation set in the article is also composed of these five parts. Set the deviation set of the information grasped by the individual and the original information *D*_*i*_ = < *d*_1*i*_, *d*_2*i*_, *d*_3*i*_, *d*_4*i*_, *d*_5*i*_ >; the deviation set of the information content diffused by the individual and the original information *S*_*i*_ = < *s*_1*i*_, *s*_2*i*_, *s*_3*i*_, *s*_4*i*_, *s*_5*i*_ >. Among them, *d*_1*i*_, *d*_2*i*_, *d*_3*i*_, *d*_4*i*_, *d*_5*i*_, *s*_1*i*_, *s*_2*i*_, *s*_3*i*_, *s*_4*i*_, *s*_5*i*_ are all described by values mapped to the interval [0,2]. *d*_1*i*_, *d*_2*i*_, *d*_3*i*_, *d*_4*i*_, *d*_5*i*_, *s*_1*i*_, *s*_2*i*_, *s*_3*i*_, *s*_4*i*_, *s*_5*i*_ <1 means negative deviation, *d*_1*i*_, *d*_2*i*_, *d*_3*i*_, *d*_4*i*_, *d*_5*i*_, *s*_1*i*_, *s*_2*i*_, *s*_3*i*_, *s*_4*i*_, *s*_5*i*_ <1 means positive deviation, *d*_1*i*_, *d*_2*i*_, *d*_3*i*_, *d*_4*i*_, *d*_5*i*_, *s*_1*i*_, *s*_2*i*_, *s*_3*i*_, *s*_4*i*_, *s*_5*i*_ = 1 means it is consistent with the original information.

##### Factors Affecting Information Interaction Among Individuals

This section quantifies the two factors that affect the information interaction between people: one is the degree of trust between individuals and the other is the individual's mastery degree of event-related information.

(1) The degree of trust between individuals. Existing research on trust theory (23–25) generally believes that “trust is the premise of information exchange between individuals and the cornerstone of social networks. If there is no interpersonal trust, social networks will collapse.” Taking the convenience of social networks into account, it can make two netizens who are not related in real life communicate, but the degree of mutual understanding of the interactive dual is not high. This leads to the fact that netizens in social networks can only determine whether to trust each other through their social influence and the strength of the relationship between netizens. Based on this, the degree of trust between individuals is determined by the individual's social influence and the strength of the relationship between them.

(1) Individual's social influence. A person's social influence refers to his/her ability to influence other ones' behaviors in a social network, and reflects the importance of an individual in the network. In complex network theory, tightness can be used as a measure of node centrality, which is defined as the average shortest path from a node to other reachable nodes. Generally, the higher the tightness is, the more important the node is. Therefore, the individual's tightness formula in the complex network is used to calculate the individual's social influence *I*_*i*_, as shown in formula (4):


(4) $$\begin{array}{l} I_{i}=\frac{N-1}{\sum_{j}s_{ij}} \end{array}$$

(2) Strength of relationships among individuals. In reality, people tend to trust their close friends more and trust the information they convey. Therefore, the strength of the relationship between individuals will have an impact on information diffusion, i.e., the closer the relationship between individuals is, the higher the degree of mutual trust is. Here, the concept of individual embedding degree (26), i.e., the number of common neighbor individuals that two individuals have in the network, is used to describe the strength of the relationship between these two individuals, as shown in formula (5):


(5) $$\begin{array}{l} R_{ij}=\{\begin{array}{ll} \frac{n_{ij}}{\left(k_{i}-1\right)+\left(k_{j}-1\right)} & k_{i},k_{j}\neq 1\\ 1 & k_{i}=k_{j}=1 \end{array} \end{array}$$

where *k*_*i*_-1 represents the number of neighbors remaining for individual *i* except for individual *j*. (*k*_*i*_-1) + (*k*_*j*_-1) represents the maximum number of common neighbor individuals that may exist between individuals *i* and *j*. In addition, the premise of setting the interaction between two entities is that they have a direct connection in the network. Therefore, when *k*_*i*_ = *k*_*j*_ = 1, it means that individuals *i* and *j* are each other's exclusive neighbors, and the relationship between the two is the strongest, i.e., *R*_*ij*_ = 1.

In summary, the calculation of the trust degree *C*_*ij*_ of individual *i* to individual *j* is shown in formula (6):


(6) $$\begin{array}{l} C_{ij}=I_{i}*R_{ij} \end{array}$$

(2) The individual's mastery of event-related information. In social networks, only a small number of people can grasp more comprehensive information, while the vast majority only grasp part of the information and make behavioral decisions based on the limited information they have. This is called information asymmetry. The phenomenon of information asymmetry is an important driving force for the rumor diffusion (27). At present, the development of the Internet has made information acquisition more and more convenient, and the asymmetry of information between individuals will be weakened. However, at the same time, it will aggravate the level of information confusion in social networks. This is because the unique free speech space of the Internet allows any information to be diffused on a large scale in a short period of time, but it costs longer time to verify the information authenticity. Therefore, although the public have more opportunities and channels to obtain information, they cannot accurately judge the authenticity of the information, which further strengthens the asymmetry of individuals in terms of information accuracy. Based on this, this article introduces the individual knowledge reserve (28) and the degree of confusion in external information (29) to describe the individual's mastery of event-related information.

(1) Individual knowledge reserves. Because most individuals do not know the true situation of public opinion events, they can only judge whether to accept external information based on their own past experience and relevant knowledge. For example, during the outbreak of COVID-19, it was widely diffused on the Internet that dual yellow oral liquid could prevent virus infection. In fact, dual yellow oral liquid cannot prevent COVID-19 virus. However, due to the lack of knowledge of pathology and virology, the public chose to believe this information, which once triggered a panic buying wave. Based on this, the individual knowledge reserve *K*_*i*_ is assumed to follow a Poisson distribution with a mean value of λ to reflect the phenomenon that only a small number of individuals in the network have a relatively professional knowledge reserve.

(2) The degree of confusion in external information. After the diffusion of information, individuals gradually are aware of information with different contents. A large amount of redundant information will interfere with their judgment of the authenticity and accuracy of the information, so that there is a greater probability of accepting rumors or rejecting real information. Here, the degree of confusion in external information Σ_*i*_ is calculated by formula (7), as follows:


(7) $$\begin{array}{l} \sum_{i}\left(t\right)=\frac{1}{5}\overset{5}{\underset{j=1}{\sum }}\left(\sqrt{\overset{n_{i}}{\underset{l=1}{\sum }}\left(d_{jl}\left(t\right)-\sum d_{jl}\left(t\right)/n_{i}\right)}/\left(n_{i}-1\right)\right) \end{array}$$

In summary, the individual's mastery of event-related information *G*_*i*_ is described by formula (8).


(8) $$\begin{array}{l} G_{i}=K_{i}*\sum_{i} \end{array}$$

##### Information Interaction Mechanism

When the information receiver has a high degree of trust in the communication individual, he/she will accept the information sent by the communication one. In addition, the communication ones are divided into ordinary communication individual O and malicious communication individual M according to diffuse intention. Among them, the ordinary communication individual diffuse information that he/she believes to be true to uninformed ones, who will not tamper or process the information in the processing of information diffusion. The malicious communication ones tamper and process the information for gaining attention and increasing influence, and diffuse processed information to others. Since the information receiving mechanism of all individuals is the same, and the information diffusion mechanism of different communication individuals is different, the information reception mechanism of the individual must be set first, and then the information diffusion mechanism of the general and the malicious communication individual must be set separately.

(1) Individual information reception mechanism

When a communication individual sends information to neighbors, the recipient of the information compares the communication individual's trust level with his/her own trust threshold at first. If the former's reputation is greater than the trust threshold, the information will be accepted by the information recipient, and vice versa. After receiving the information, the information recipient updates the content that he/she believes to be true according to his/her mastery of the event-related information. The specific reception mechanism is as follows:

When *C*_*ij*_ ≥ *c*_*i*_


(9) $$\begin{array}{c} d_{1i}\left(t+1\right)=d_{1i}\left(t\right)+G_{i}\left(s_{1j}\left(t\right)-d_{1i}\left(t\right)\right)\\ d_{2i}\left(t+1\right)=d_{2i}\left(t\right)+G_{i}\left(s_{2j}\left(t\right)-d_{2i}\left(t\right)\right)\\ d_{3i}\left(t+1\right)=d_{3i}\left(t\right)+G_{i}\left(s_{3j}\left(t\right)-d_{3i}\left(t\right)\right)\\ d_{4i}\left(t+1\right)=d_{4i}\left(t\right)+G_{i}\left(s_{4j}\left(t\right)-d_{4i}\left(t\right)\right)\\ d_{5i}\left(t+1\right)=d_{5i}\left(t\right)+G_{i}\left(s_{5j}\left(t\right)-d_{5i}\left(t\right)\right)\\ d_{i}\left(t+1\right)=\langle d_{1i}\left(t+1\right),d_{2i}\left(t+1\right),d_{3i}\left(t+1\right),d_{4i}\left(t+1\right),d_{5i}\left(t+1\right)\rangle \end{array}$$

When *C*_*ij*_ < *c*_*i*_


(10) $$\begin{array}{l} d_{i}\left(t+1\right)=d_{i}\left(t\right)=\langle d_{1i}\left(t\right),d_{2i}\left(t\right),d_{3i}\left(t\right),d_{4i}\left(t\right),d_{5i}\left(t\right)\rangle \end{array}$$

(2) Information diffusion mechanism of ordinary communication individuals

Generally speaking, while diffusing information to the outside world, ordinary communication individuals will directly diffuse the information they believe to be true to other individuals, i.e.,


(11) $$\begin{array}{l} s_{i}\left(t+1\right)=d_{i}\left(t+1\right)=\langle d_{1i}\left(t+1\right),\\ \;d_{2i}\left(t+1\right),d_{3i}\left(t+1\right),d_{4i}\left(t+1\right),d_{5i}\left(t+1\right)\rangle \end{array}$$

(3) Information diffusion mechanism of malicious communication individuals

Before diffusing information externally, malicious communication individuals will tamper with the information they believe to be true to a certain extent, and diffuse the tampered information to others. The degree of tampering will increase with the increase of the gain from the feedback of the tampered information, and decrease with the increase of the punishment of rumors. Therefore, the formula for calculating the tampered intensity Δ_*i*_ is as follows:


(12) $$\begin{array}{l} \Delta_{i}\left(t\right)=\text{ln}\left(e^{1-p}-\frac{1}{m_{it}}\right) \end{array}$$

Information diffusion mechanism of malicious communication individuals is as follows:


(13) $$\begin{array}{c} s_{1i}\left(t+1\right)=d_{1i}\left(t+1\right)*\left(1+{\left(-1\right)}^{\beta }\Delta_{i}\left(t+1\right)\right)\\ s_{2i}\left(t+1\right)=d_{2i}\left(t+1\right)*\left(1+{\left(-1\right)}^{\beta }\Delta_{i}\left(t+1\right)\right)\\ s_{3i}\left(t+1\right)=d_{3i}\left(t+1\right)*\left(1+{\left(-1\right)}^{\beta }\Delta_{i}\left(t+1\right)\right)\\ s_{4i}\left(t+1\right)=d_{4i}\left(t+1\right)*\left(1+{\left(-1\right)}^{\beta }\Delta_{i}\left(t+1\right)\right)\\ s_{5i}\left(t+1\right)=d_{5i}\left(t+1\right)*\left(1+{\left(-1\right)}^{\beta }\Delta_{i}\left(t+1\right)\right)\\ s_{i}\left(t+1\right)=\langle s_{1i}\left(t+1\right),s_{2i}\left(t+1\right),s_{3i}\left(t+1\right),s_{4i}\left(t+1\right),s_{5i}\left(t+1\right)\rangle \end{array}$$

where β is a random number of either 0 or 1.

#### Framework and Simulation Steps of SEIR-OM Model

**Figure 4:**
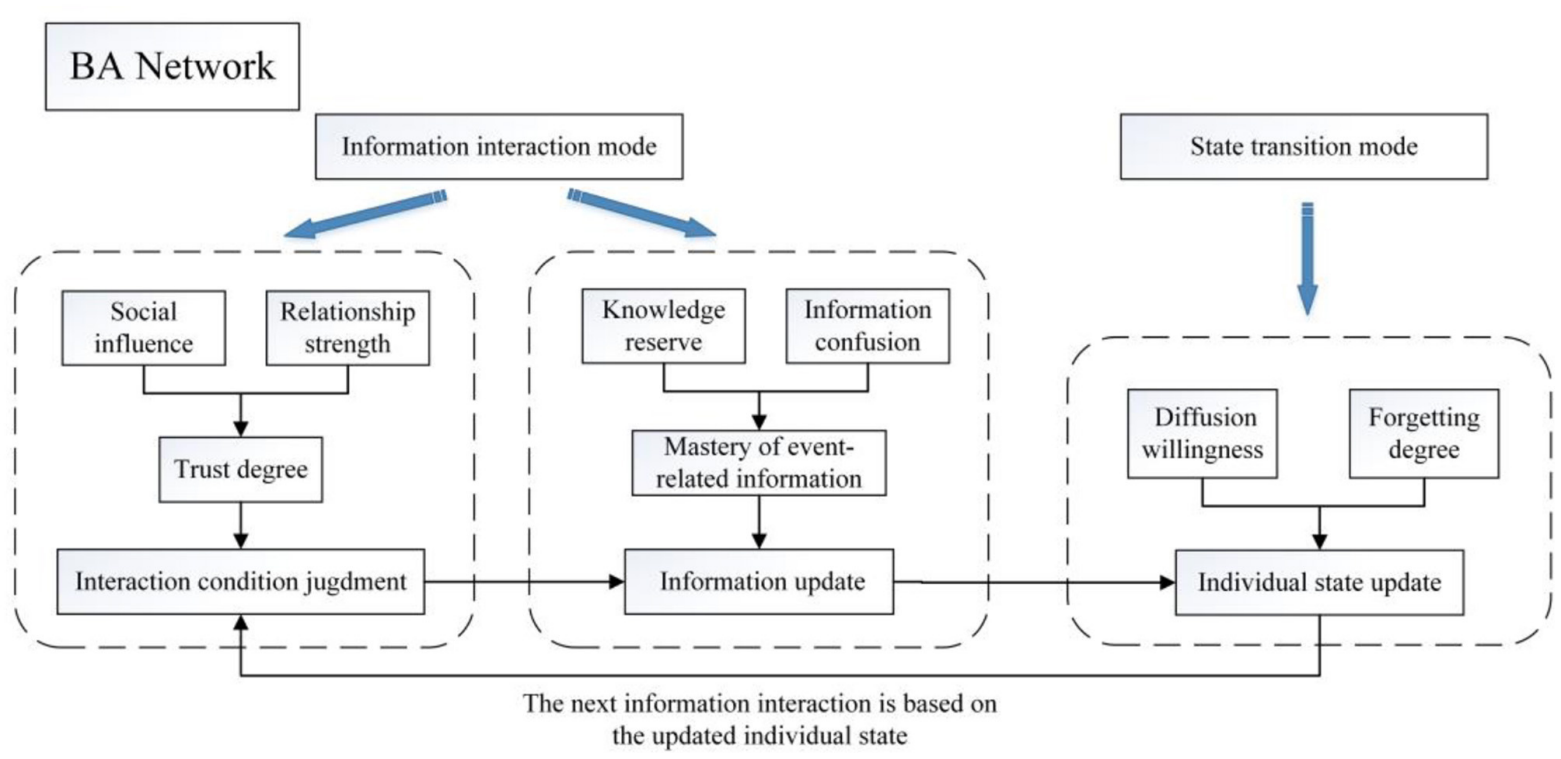
Construction of SEIR-OM model.

Based on the Barabási-Albert scale-free network (BA network) (30, 31), the Monte Carlo simulation method based on multi-agent is used to simulate the SEIR-OM model. Its construction process is shown in Figure 4.

Compared with the classical SEIR model, the state transition mode in SEIR-OM model can more reasonably and carefully describe the psychological mechanism of individual state transition. The information interaction mode in SEIR-OM model can not only distinguish different information contents, but also reflect the individual's psychological decision before receiving (transmitting) information.

The specific process of the formation and diffusion of rumors is as follows:

(1) At the initial moment, a certain number of malicious communication individuals and general communication individuals are randomly generated, and their initial diffusion willingness and the forgetting degree of public opinion events are generated according to formulas (1) and (2), respectively.

(2) At any time, the communication individual *i* randomly selects its neighbor individual *j* as the object of information interaction. If the trust degree of *i* is greater than or equal to the trust threshold of *j*, information interaction is carried out according to the state of *j*. Generally, there are the following two situations: (1) If *j* is an uninformed individual, he/she will fully accept the information diffused by *i*, form the initial diffusion willingness and the initial forgetting degree, and transform it into a communication individual or a silent individual according to the initial diffusion willingness. (2) When *j* is a silent individual or a communication individual, the communication individuals *i* and *j* exchange information according to the formulas (9–13). If the trust degree of the communication individual *i* is less than the trust threshold of *j*, they will not exchange information.

(3) At any time, after all communication individuals have completed their outward communication, they update the individual's diffusion willingness, forgetting degree, and individual state in the network.

(4) Determine whether the end condition is met. The conditions for ending the interaction are set as follows:


(14) $$\begin{array}{l} \frac{\overset{N}{\underset{i=1}{\sum }}v_{i}\left(t\right)}{N}\leq 0.1 \end{array}$$

**Figure 5:**
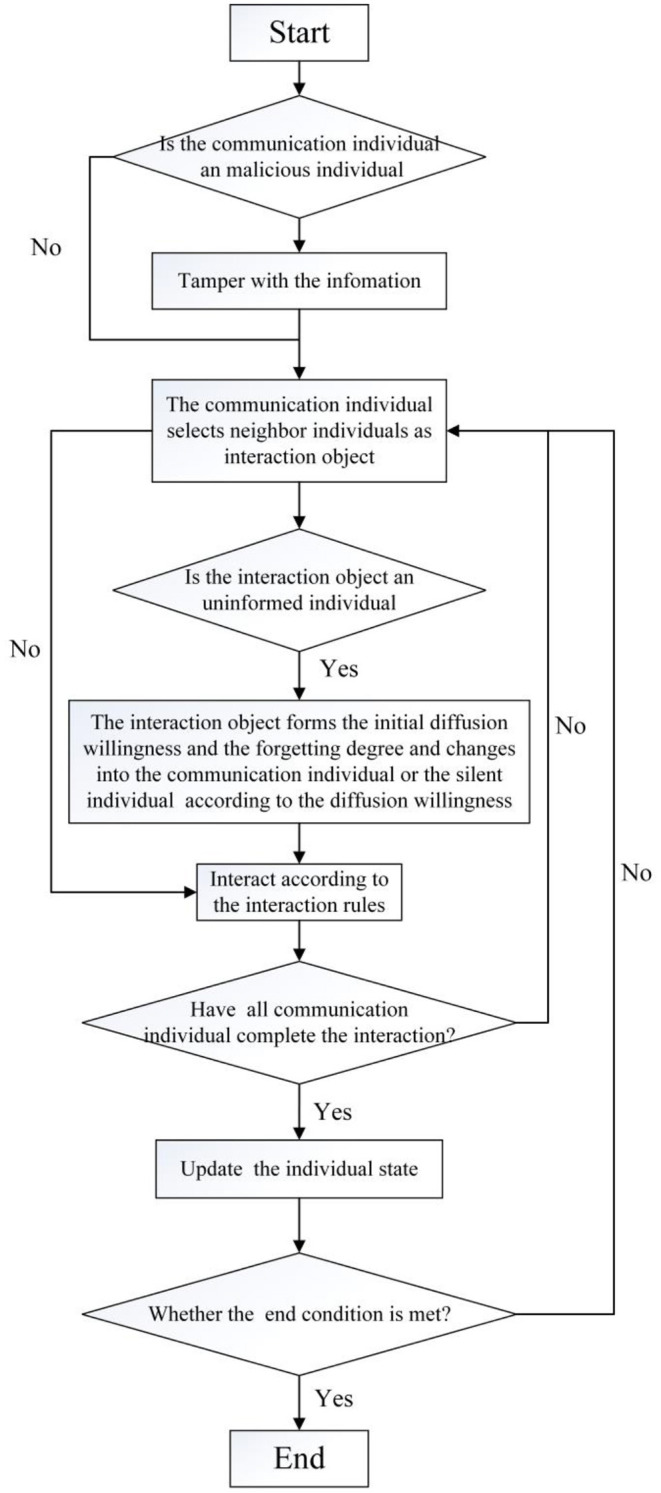
Simulation flow of the formation and diffusion of rumors.

(5) If the interaction end condition is not satisfied, repeat steps (2)–(4) until formula (14) is satisfied, and the interaction process ends. The specific process is shown in Figure 5.

## Simulation Experiment

This section uses the Monte Carlo simulation method based on multi-agent system to explore the influences of model parameters on the process of rumor diffusion and the implementation effects of different rumor control strategies. The simulation network is constructed with BA network, and the individual scale in the network is set to 300.

### The Influence of Model Parameters on the Process of Rumor Diffusion

This section starts with the model parameters and analyzes its influence on the diffusion process of rumors. There are 2 comparison indicators used in the analysis:

(1) Entire network information content deviation: it refers to the average value of the deviation between the information content in the network and the actual information content. Here, the deviation *dev*_*i*_(*t*) between the information content diffused by the individual *i* and the real information content is set. The calculation is shown in formula (15), and the calculation of the deviation of the entire network information content deviation is shown in formula (16).


(15) $$\begin{array}{lll} dev_{i}\left(t\right) & = & \frac{\sqrt{\overset{5}{\underset{x=1}{\sum }}{\left(s_{xi}\left(t\right)-1\right)}^{2}}}{5} \end{array}$$

(16) $$\begin{array}{lll} Deviation\left(t\right) & = & \frac{\overset{N}{\underset{i=1}{\sum }}dev_{i}\left(t\right)}{N} \end{array}$$

(2) Rumor diffusion range: it refers to the proportion of individuals holding rumors in the network to the total number of individuals on the network. Here, information with content deviation >0.5 is identified as a rumor, and the calculation is shown in formula (17). Based on this, the calculation of the rumor diffusion range is shown in formula (18):


(17) $$\begin{array}{l} d_{i}\left(t\right)\leftarrow \{\begin{array}{c} \text{rumor}\\ \text{truth} \end{array}\begin{array}{l} \text{ if }dev_{i}\left(t\right)\geq 0.5\\ \text{ if }dev_{i}\left(t\right)<0.5 \end{array} \end{array}$$

(18) $$\begin{array}{l} Breath\left(t\right)=\frac{\sum \text{rumor}}{N} \end{array}$$

#### The Impact of μ_*b*_ on Rumor Evolution Process

**Figure 6:**
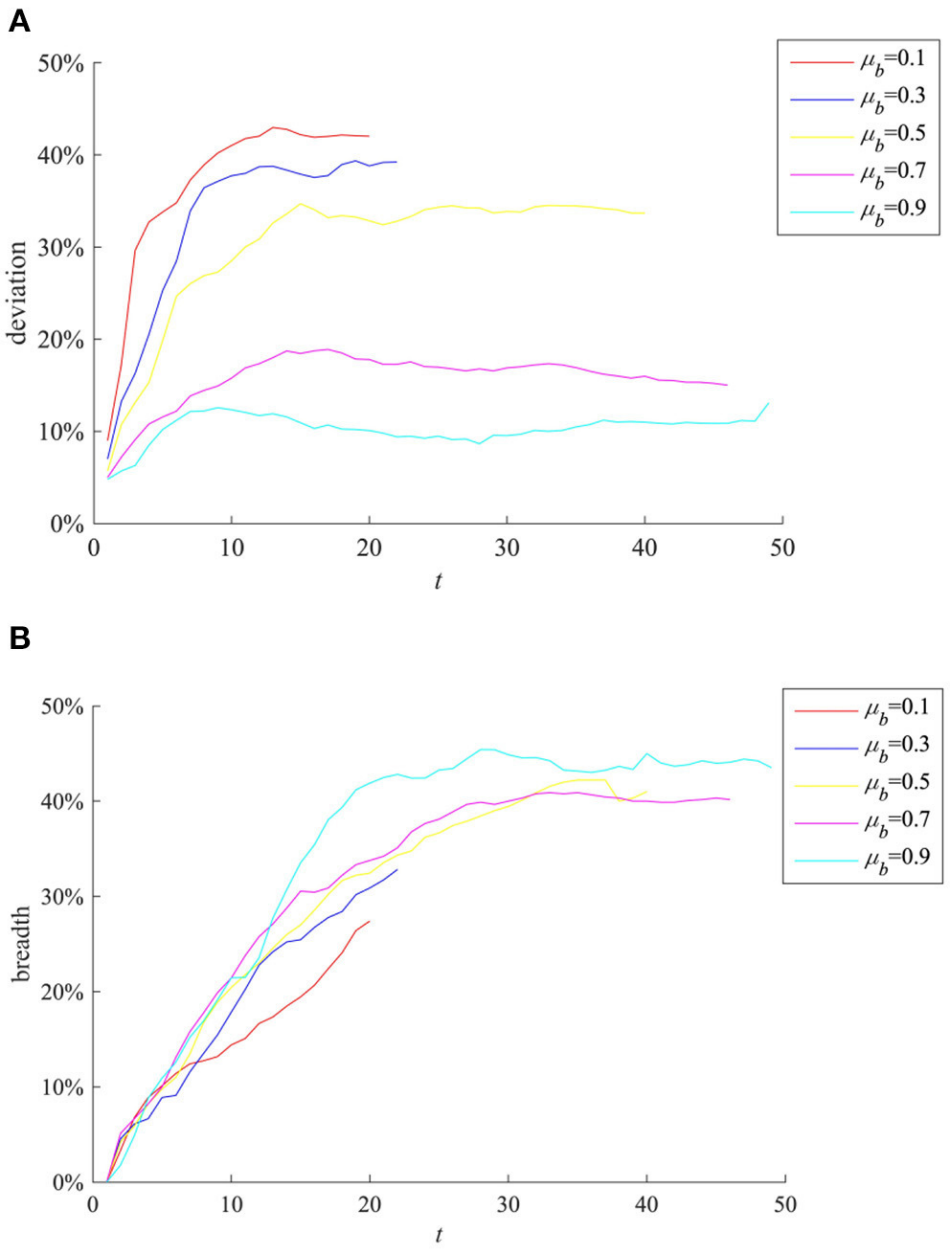
The impact of μ_*b*_ on rumor evolution process. **(A)** Entire network information content deviation. **(B)** Rumor diffusion range.

The mean value of the degree of interest correlation between all individuals and public opinion events μ_*b*_ will affect the individual's attention to the event, and thus have an impact on the diffusion of event-related information. Here take μ_*b*_ as 0.1, 0.3, 0.5, 0.7, and 0.9, respectively, for comparison. The results are shown in Figure 6.

It can be seen from Figure 6A that as μ_*b*_ increases, the entire network information content deviation decreases. This shows that when the event is related to the interests of most individuals, they are more concerned about the authenticity of the information and more cautious about the information sent by the outside world, so that the entire network information content deviation of the entire network is lower. It can be seen from Figure 6B that as μ_*b*_ is larger, the rumor costs longer time to diffuse and its diffusion range is wider. This shows that individuals are more concerned about the incident and have a stronger willingness to forward information related to their own interests, and protect their own interests by expanding the influence of the incident, which also provides opportunities for the rumor diffusion and makes more widespread.

#### The Impact of Network Structure on Rumor Evolution Process

**Figure 7:**
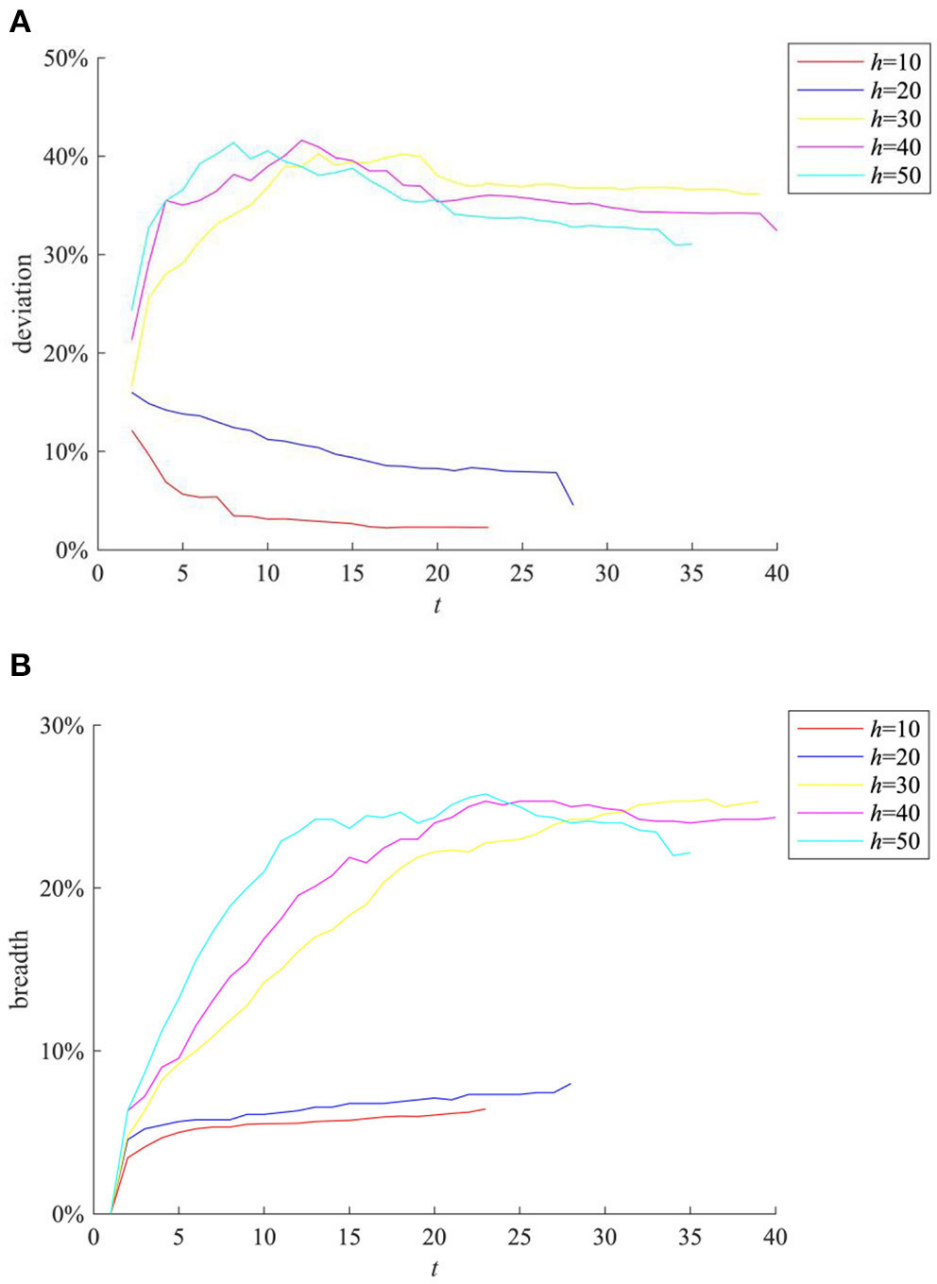
The impact of network structure on rumor evolution process. **(A)** Entire network information content deviation. **(B)** Rumor diffusion range.

Social networks provide channels for information diffusion. If the network structure changes, the strength of relationships among individuals will change accordingly, which will affect the reception and diffusion of information. In order to study the influences of different network structures on the process of rumor diffusion, this section changes the value of *h* (*h* ∈ [0,*N*]) (Note that the BA network used in the simulation experiment is based on an interconnected network, after the introduction of several new nodes. The new nodes will be connected to *h* existing nodes). Our experiments generate BA networks of different structures, and compare the rumor diffusion under different network structures, then the results are as shown in Figure 7.

It can be seen from Figures 7A,B that when *h* = 20, the information content deviation of the entire network and the rumor diffusion range are higher than the case of *h* = 10. When *h* = 30, the information content deviation of the entire network exceeds 30%, and the rumor diffusion range also exceeds 20%. It can be seen that the increase of *h* can promote the rumor diffusion. This is mainly because when *h* is small, the average degree of network nodes is low, and the connection between individuals is weak, which makes the information diffusion channel blocked, resulting in small rumor diffusion range. With the increase of *h*, the average network degree of nodes increases, the connection among individuals is strengthened, and the information interactions among individuals become more frequent, which creates conditions for the rumor diffusion. However, it is easy to find that when *h* ≥ 30, the increase of *h* no longer expands the entire network information content deviation of the entire network and the rumor diffusion range, indicating that the average network degree of nodes has a peak in the influence of rumor diffusion range.

#### The Impact of μ_*c*_ on Rumor Evolution Process

**Figure 8:**
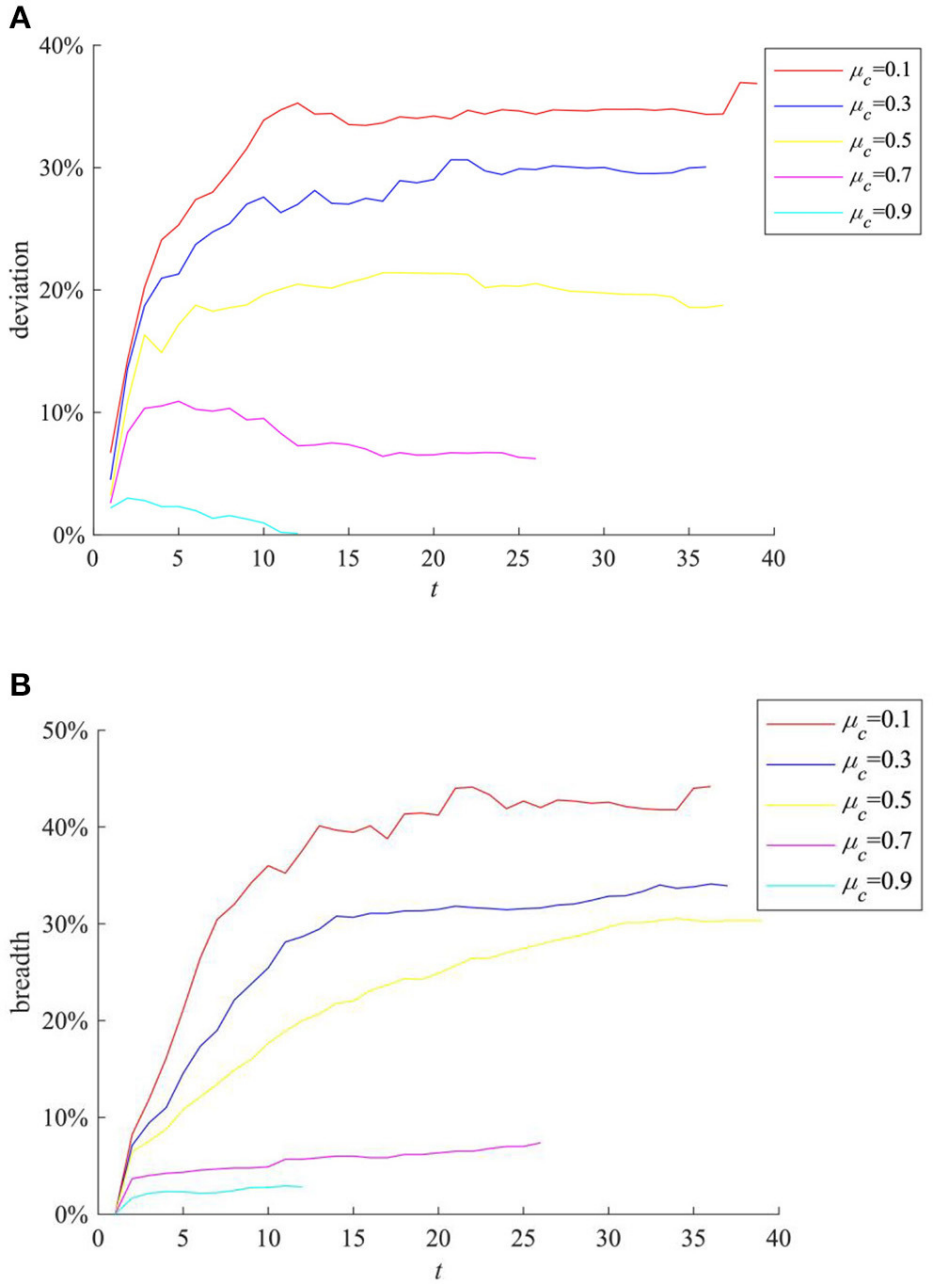
The impact of μ_*c*_ on rumor evolution process. **(A)** Information content deviation. **(B)** Rumor diffusion range.

The trust threshold reflects the cautious of an individual treating external information, and its value will affect his/her reception of external information. Here we select the cases where the mean value of individual trust threshold μ_*c*_(μ_*c*_ = ∑ (*c*_*i*_)/*N*) is 0.1, 0.3, 0.5, 0.7, 0.9, respectively, for comparison, and the results are shown in Figure 8.

It can be seen from Figures 8A,B that with the increase of μ_*c*_, the entire network information content deviation and the rumor diffusion range gradually decrease. This is because the increase in the average trust threshold means that the information recipients generally reduce their trust in the network, and they are increasingly inclined to refuse to information from the outside world, resulting in more obstacles for information diffusion, making it impossible for further diffusion.

### Analysis of Rumor Control Strategy

In this section, according to the time and means of implementing the rumor control strategies, they are divided into proactive prevention and reactive rumor refuting ones, as shown in Table 3. Among them, the prevention strategy refers to the preventive strategy taken before the occurrence of public opinion incidents. The reactive strategy refers to the refuting strategy taken after the occurrence of the rumors. Here, the effects of the two types of strategies are compared and analyzed through simulation experiments.

**Table 3:** Rumor control strategy classification.

**Classification**	**Time**	**Measure**
Proactive prevention strategy	Before the occurrence of public opinion incidents	Popularize relevant knowledge and improve the public's ability to discern rumors; establish a punishment mechanism to punish the communicator of rumors.
Reactive rumor-refuting strategy	After the occurrence of public opinion incidents	Organize some individuals to refute rumors

The current academic research on rumor control mainly focuses on how to reduce the impact of rumors, and rarely considers the negative impact of rumor control strategies, which leads to insufficient network activity. Currently, the social network has become an important channel for the media to release information, the public to obtain information, and the public to seek appeals. Insufficient network activity will prevent the important information from being diffused, and it will not satisfy the public's right to know public events. Based on this, the number of individuals participating in information interaction at different time moments is calculated as a measure of network activity to reflect the changes in network activity under different control strategies, so as to more comprehensively compare and analyze the positive and negative effects of different rumor control strategies.

#### Proactive Prevention Strategy

According to the different implementation methods of the strategy, the proactive prevention strategy is further divided into the knowledge popularization strategy and the punishment and restriction strategy.

(1) Knowledge popularization strategy

**Figure 9:**
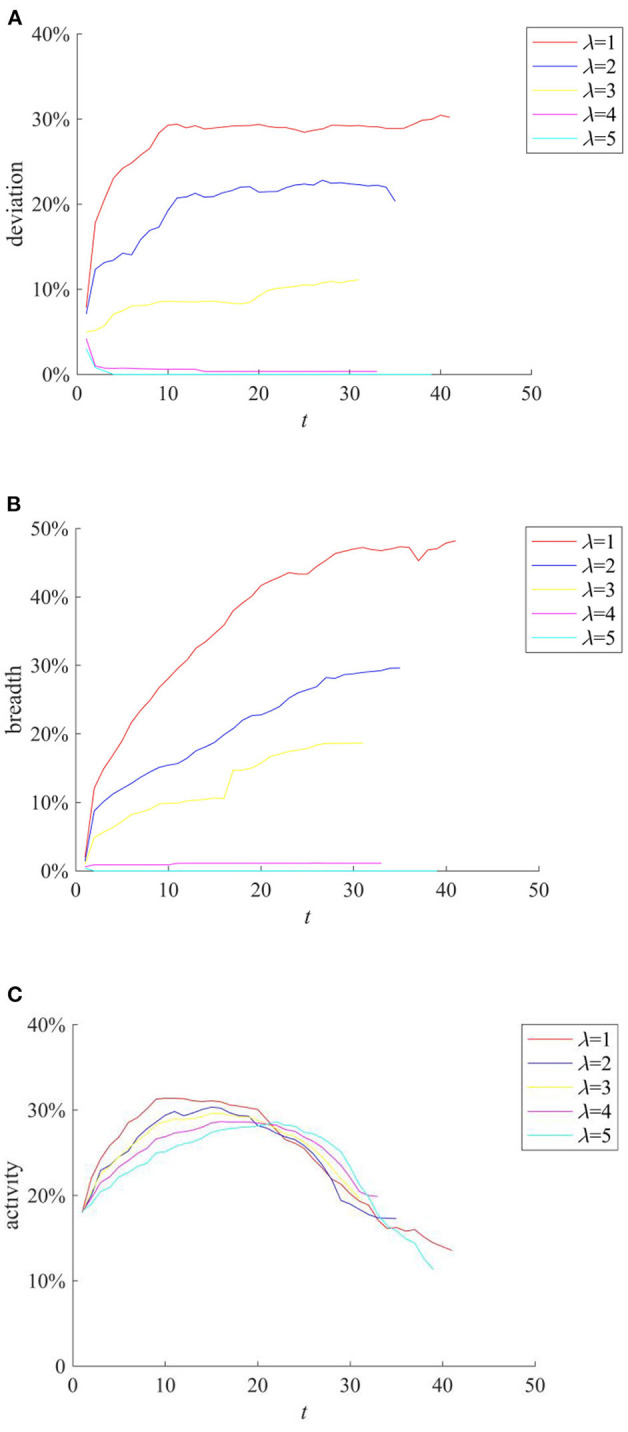
The effects of different knowledge popularization strategies. **(A)** Entire network information content deviation. **(B)** Rumor diffusion range. **(C)** Network activity.

The knowledge popularization strategy refers to the one to restrict rumor diffusion by popularizing relevant knowledge in the field to individuals before the occurrence of public opinion events in a certain field. Here, the average knowledge reserves of network individuals reflect the implementation of the knowledge popularization strategy. They are set to follow the Poisson distribution with the mean λ of 1, 2, 3, 4, and 5, respectively, and the rumor diffusion when individuals have different levels of knowledge reserves is compared. The results are shown in Figure 9.

It can be seen from Figures 9A,B that with the increase of λ, the higher level of individual knowledge reserves in the network represents the greater probability of the authentic identification information and greater possibility of rejecting rumors, reducing the scale of information content deviation and rumor diffusion range. It can be seen that adopting knowledge popularization strategies can effectively reduce the influence of rumors. In addition, it can be seen from Figure 9C that with the increase of λ, the peak value of network activity decreases, but its descend range is smaller. This is because when individual knowledge reserves are small, the public knows less about the causes and consequences of public events. In order to satisfy their own curiosity, they often trigger large-scale discussions on the Internet. However, with the increase of individual knowledge reserves, individuals can reason and derive the causes and consequences of events based on their own knowledge, which reduces the discussion on the network, and decreases network activity.

(2) Punishment and restriction strategy

**Figure 10:**
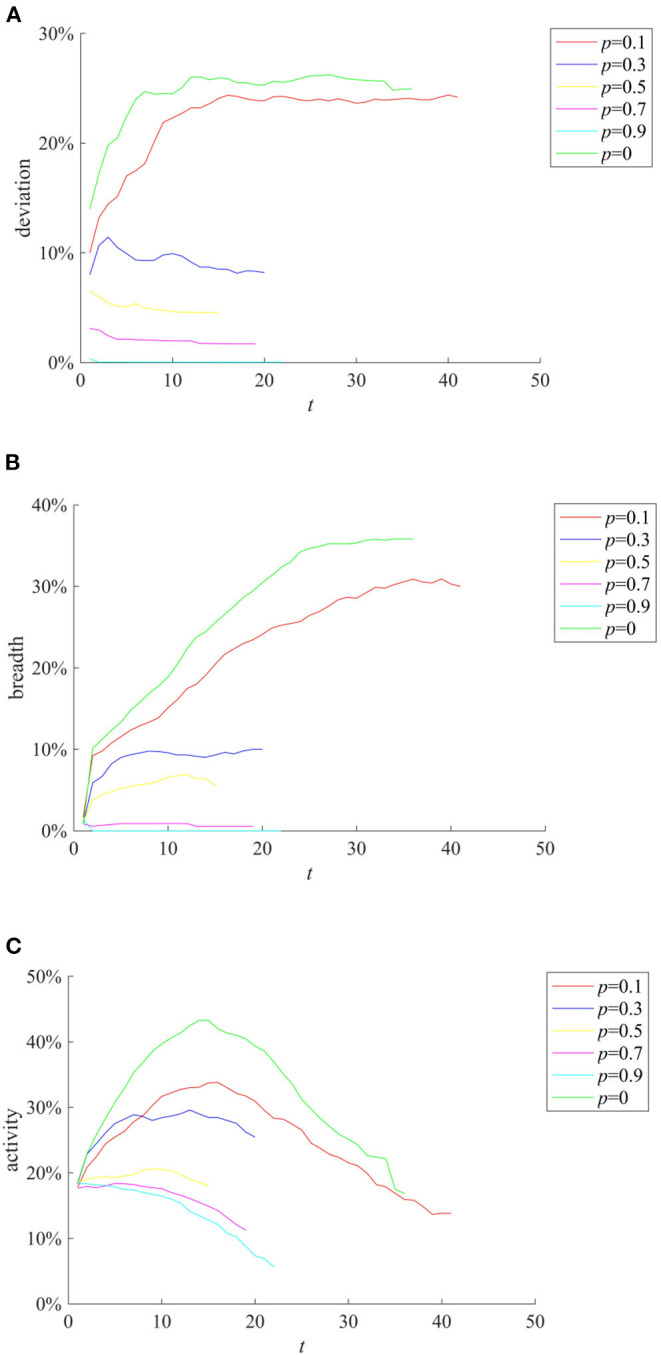
The effects of different punishment and restriction strategies. **(A)** Entire network information content deviation. **(B)** Rumor diffusion range. **(C)** Network activity.

Punishment and restriction strategy refers to the establishment of online code of conduct and punishment mechanism before the occurrence of public opinion incidents to restrict the individual behavior and rumor diffusion. Here we compare the rumor diffusion when the government punishment is 0.1, 0.3, 0.5, 0.7, and 0.9. The results are shown in Figure 10.

It can be seen from Figures 10A–C that as the punishment *p* increases, the entire network information content deviation range, the rumor diffusion, and the network activity decrease accordingly. In addition, when *p* = 0.1, the information content deviation has decreased, but it is close to the situation when there is no punitive measures. When *p* is equal to 0.5, 0.7, and 0.9, respectively, although the information content deviation and the rumor diffusion range are very low, the network activity is insufficient. In contrast, when *p* = 0.3, while avoiding low network activity, the entire network information content deviation and the rumor diffusion range are well-controlled.

(3) Analysis of combined proactive strategies

**Figure 11:**
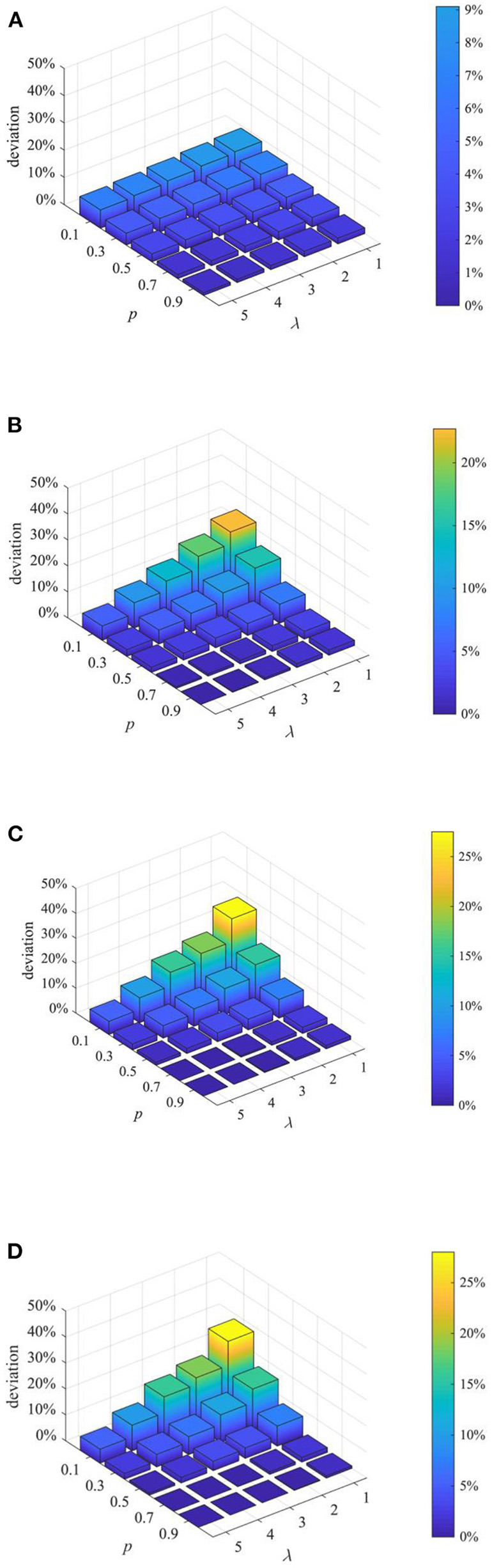
The entire network information content deviation under different strategy combinations. **(A)** *t* = 1. **(B)** *t* = 15. **(C)** *t* = 30. **(D)** *t* = 45.

**Figure 12:**
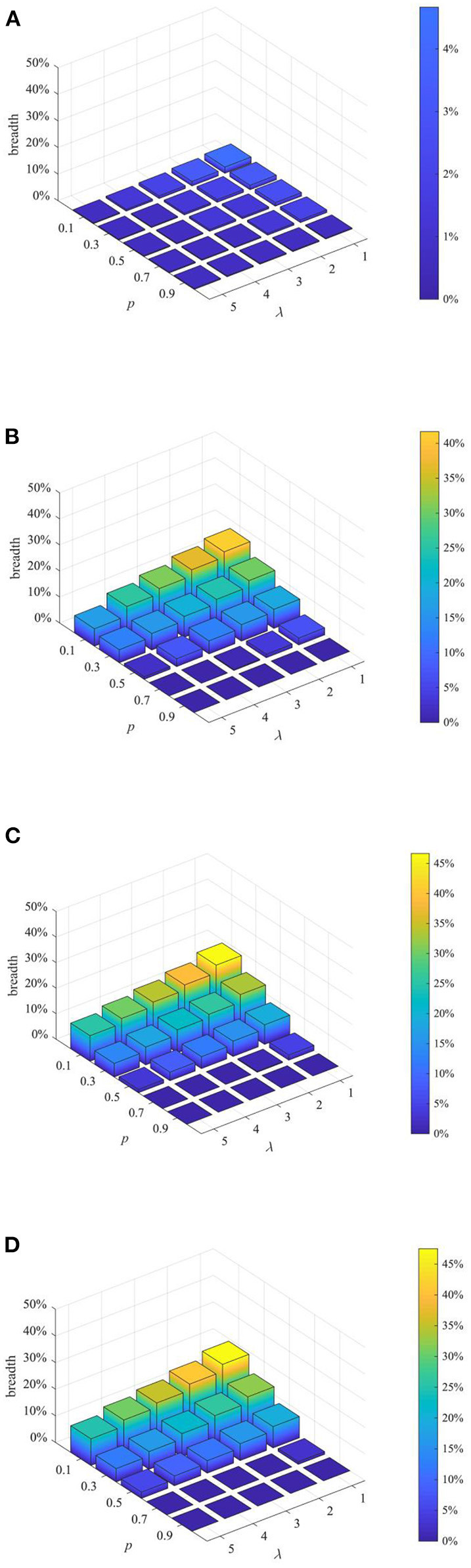
Rumor diffusion range under different strategy combinations. **(A)** *t* = 1. **(B)** *t* = 15. **(C)** *t* = 30. **(D)** *t* = 45.

**Figure 13:**
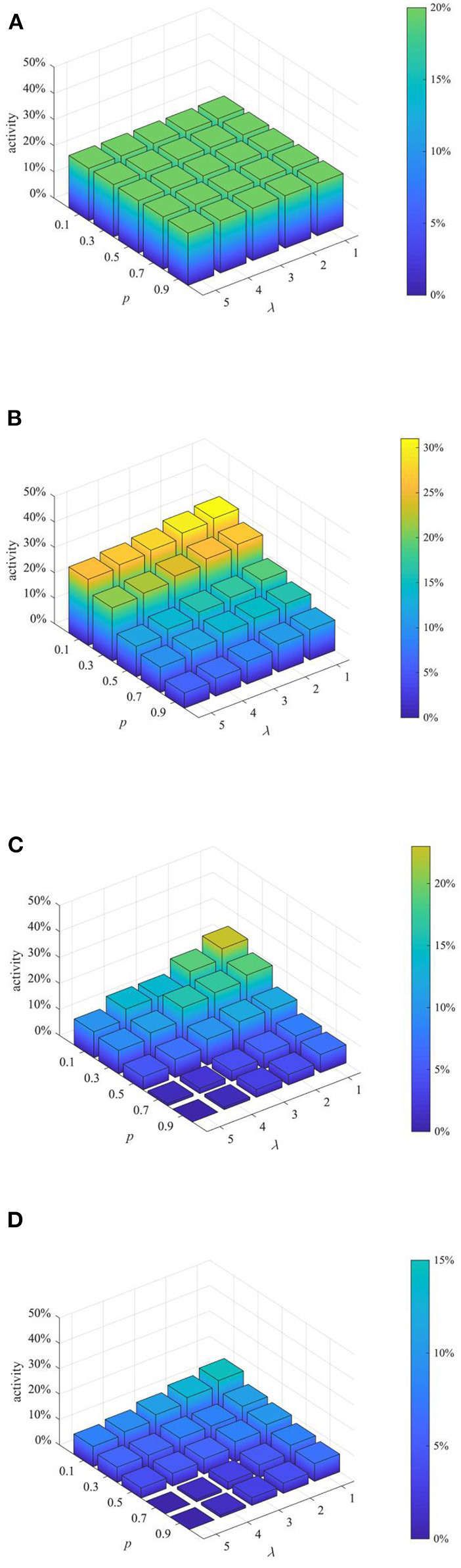
Network activity under different strategy combinations. **(A)** *t* = 1. **(B)** *t* = 15. **(C)** *t* = 30. **(D)** *t* = 45.

After analyzing the above two proactive strategies separately, this section analyzes the different combined effects of the two strategies. Here, set *p* equal to 0.1, 0.3, 0.5, 0.7, 0.9, and λ equal to 1,2 3, 4, and then combine *p* and λ with different values to form different strategy combinations, and compare the effects of different strategy combinations at *t* = 1, 15, 30, and 45. The results are shown in Figures 11–13.

It can be seen from Figures 11–13 that with the increase of *p* and λ, the entire network information content deviation, the rumor diffusion range, and the network activity are continuously reduced. In addition, when λ is fixed, with the increase of *p*, the information content deviation, network activity and the rumor diffusion will be significantly reduced. When *p* is fixed, with the increase of λ, the decrease in network activity will be smaller, and the rumor diffusion range will be slightly reduced. Although the information content deviation of the entire network is greatly reduced, the rate of decrease is relatively slow. It can be seen that the rumor control effect of the punishment and restriction strategy is better than that of the knowledge popularization one, but its restraining influence on the network activity is also far greater than that of the knowledge popularization strategy.

#### Reactive Strategy

After the rumors are formed, it is necessary to adopt a strategy of dispelling the rumors to suppress the rumor diffusion. In general, the basic idea of the rumor rejection strategy is as follows: when the rumors diffuse to a certain extent, a certain number of nodes are randomly selected as the rumor-refuting individuals, which will diffuse real information to other nodes at a certain frequency, and finally achieve the effect of suppressing the rumors.

**Figure 14:**
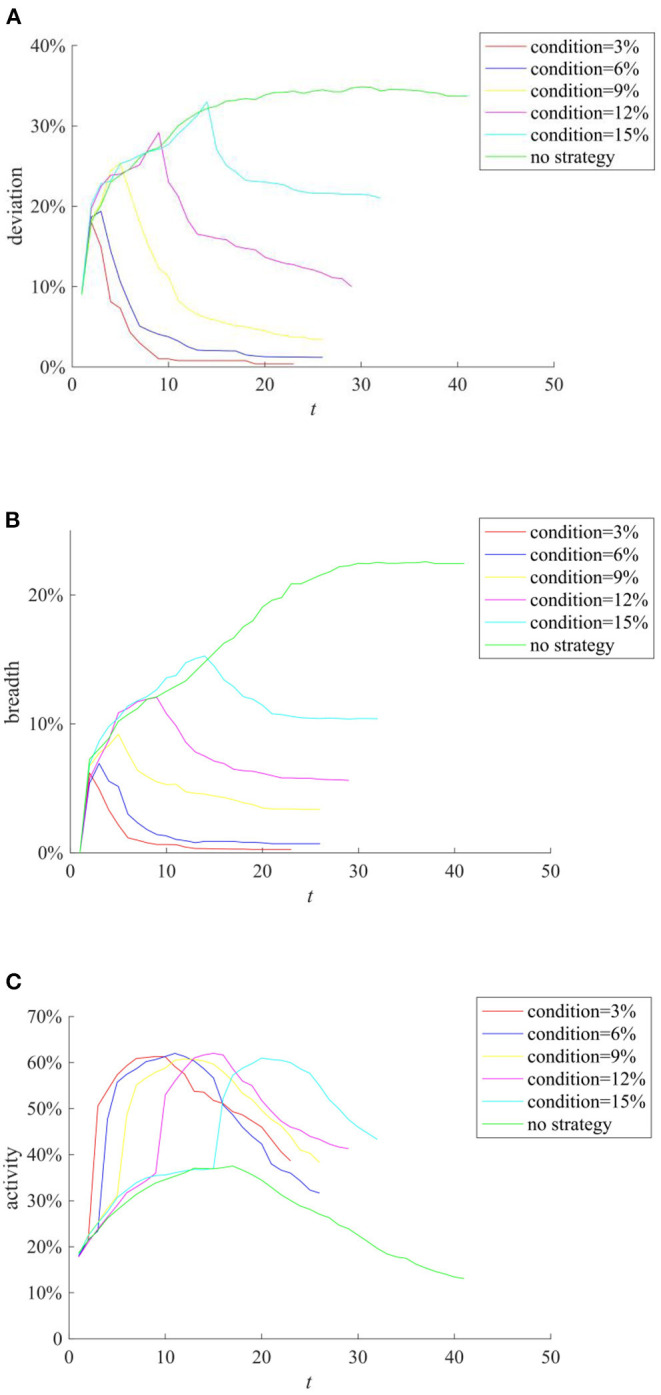
The influence of the time moment on the effect of rumor refuting strategy. **(A)** Entire network information content deviation. **(B)** Rumor diffusion range. **(C)** Network activity.

Here, we first compare the influence of the time moment on the effect of rumor refuting strategy. Figure 14 compares the implementation effects of selecting the same number of network nodes as rumor-refuting individuals when the rumors diffusion range reaches 3, 6, 9, 12, and 15%, and disseminating rumors at the same frequency.

It can be seen from Figure 14 that there are significant differences between the entire network information content deviation and the rumor diffusion range under the rumor refuting strategy. After adopting rumor-refuting strategy, the entire network information content deviation and the rumor diffusion range immediately changed from a rapid rise to a rapid decline. It can be seen from Figures 14A–C that when the rumor diffusion range reaches 3 or 6%, the rumor refuting strategy can quickly reduce the influence of rumors in a short period of time, purify relevant network information content, and finally make the rumors almost disappear. Although adopting a strategy to refute rumors when the scale of rumor diffusion range reaches 9, 12, and 15% can also greatly reduce the impact of rumors, as the time moment of the strategy is postponed, the rumors have formed a certain scale and the difficulty of refuting rumors has increased. The final effect of the strategy gradually deteriorated. In addition, the adoption of rumor refuting strategy has greatly increased network activity, and has caused a new round of useful discussions on public opinion events. To sum up, after a public opinion incident occurs, the government should adopt a rumor-refuting strategy as soon as possible to minimize the impact of rumors.

**Figure 15:**
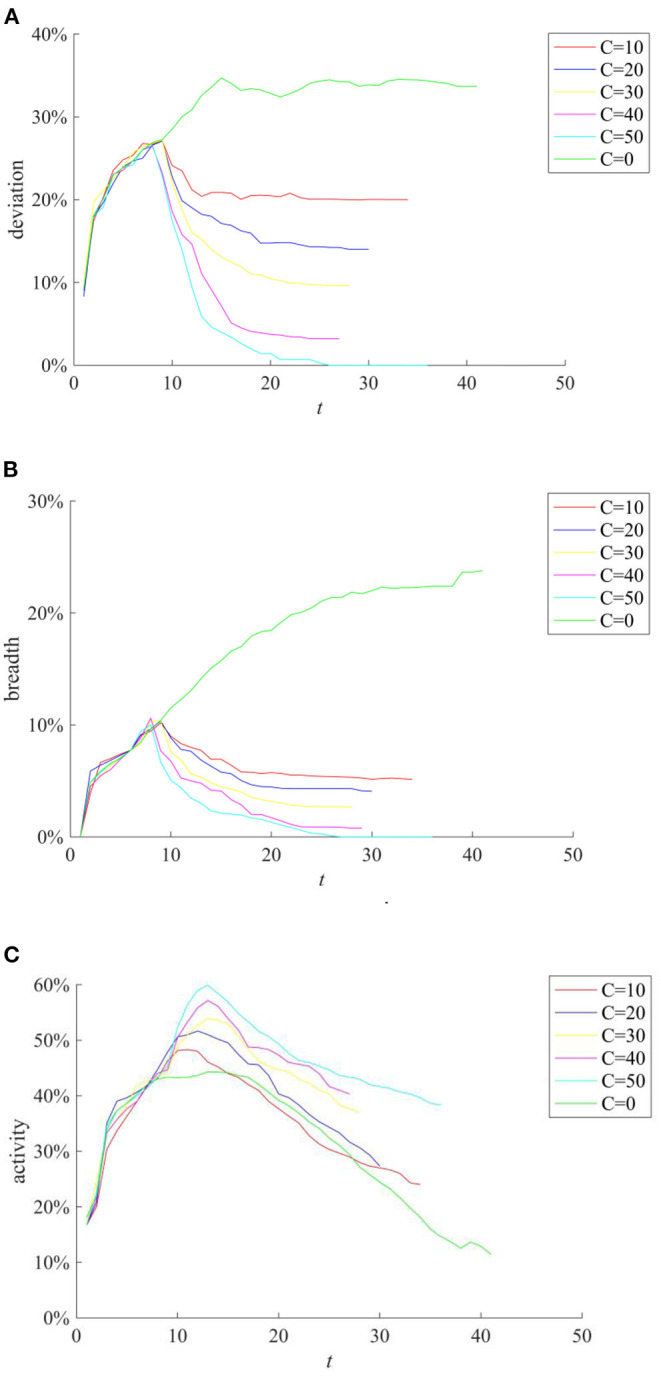
The effect of rumor-refuting strategies based on different rumor-refuting individuals. **(A)** Information content deviation. **(B)** Rumor diffusion range. **(C)** Network activity.

**Figure 16:**
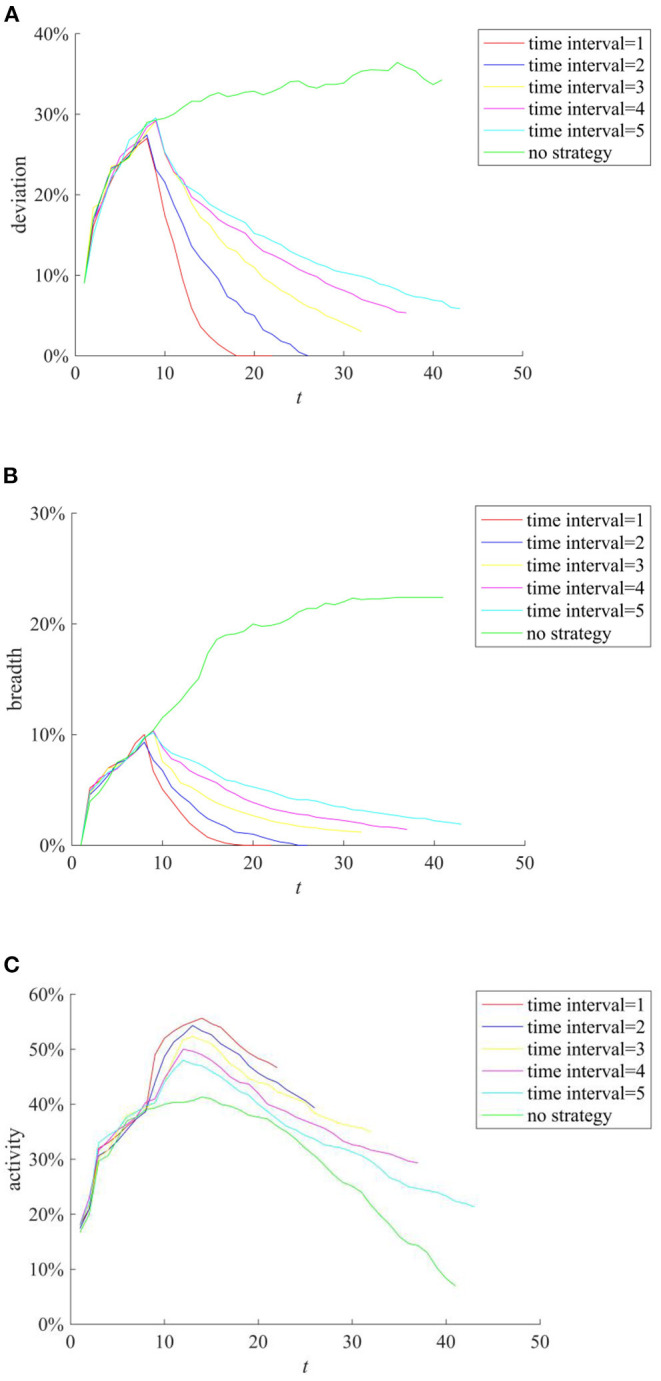
The effect of rumor-refuting strategies based on different rumor time refuting interval. **(A)** Entire network information content deviation. **(B)** Rumor diffusion range. **(C)** Network activity.

In addition, during the implementation of the rumor-refuting strategy, the time interval of rumor-refuting (the time interval between two adjacent rumor-refuting behaviors) and the number of individuals that refute the rumors (the individuals that refute the rumors point to other individuals who diffuse the true information content) will affect the ultimate effect of the rumor-refuting strategy. Figure 15 compares the implementation effects of selecting 10, 20, 30, 40, and 50 network nodes as individuals to dispel rumors when the rumor diffusion reaches 10%. Figure 16 compares the implementation effect of selecting the same number of network nodes as the rumor-refuting individuals and diffusing the rumor-refuting information at intervals of 1, 2, 3, 4, and 5 when the rumor diffusion reaches 10%.

It can be seen from Figures 15A,B that with the increase of the number of rumor-refuting individuals, the scale of entire network information content deviation and rumor diffusion range has dropped significantly. In addition, it can be seen from Figures 16A,B that the shorter the time interval of refuting rumors, the more effective the rumor-refuting strategy will be. Moreover, from Figures 15C, 16C, it can be seen that the increase in the number of rumor-refuting individuals and the expansion of the time interval for rumor-refuting have significantly improved network activity.

**Figure 17:**
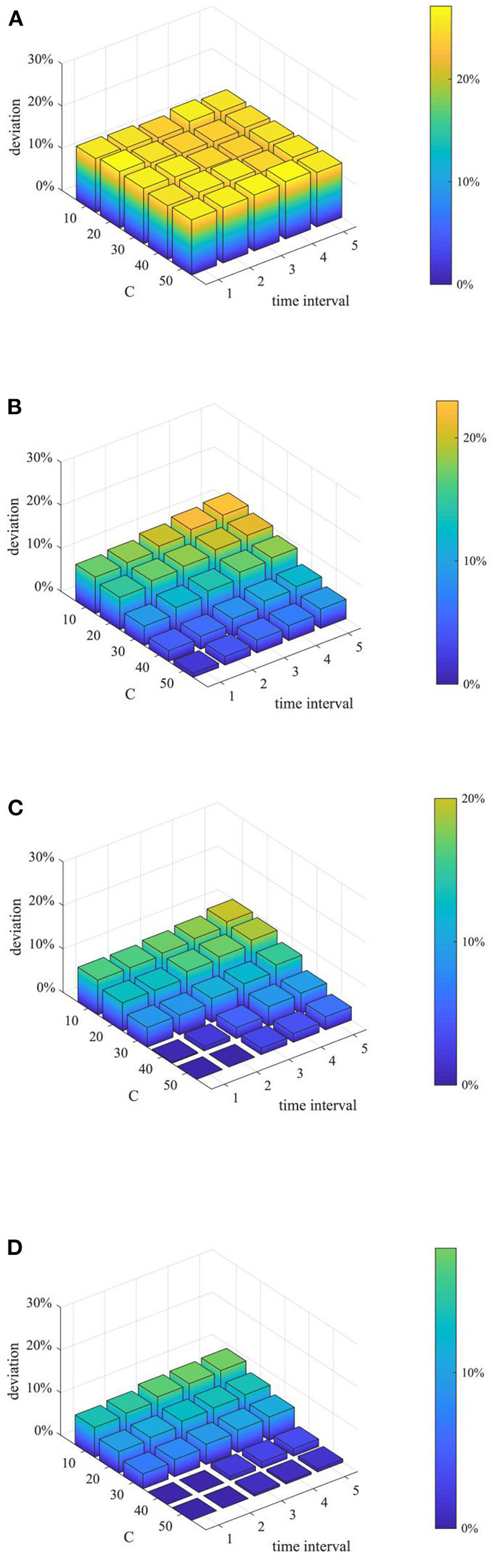
Entire network information content deviation based on different combination. **(A)** *t* = 10. **(B)** *t* = 20. **(C)** *t* = 30. **(D)** *t* = 40.

**Figure 18:**
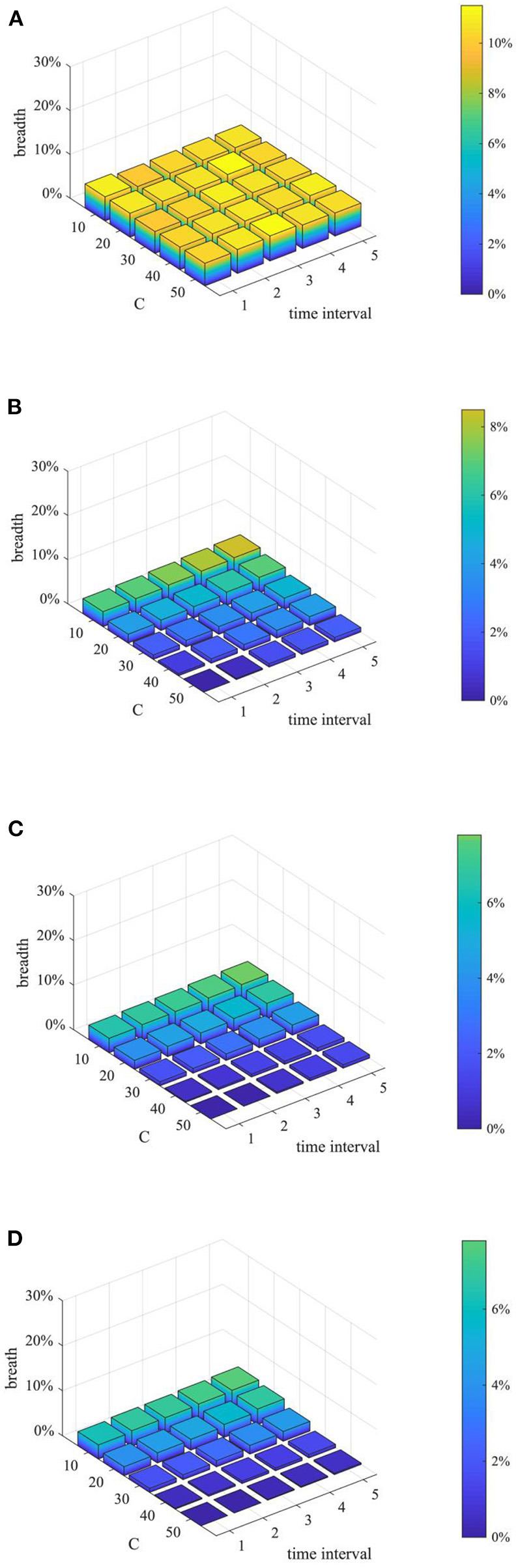
Rumor diffusion range based on different combination. **(A)** *t* = 10. **(B)** *t* = 20. **(C)** *t* = 30. **(D)** *t* = 40.

**Figure 19:**
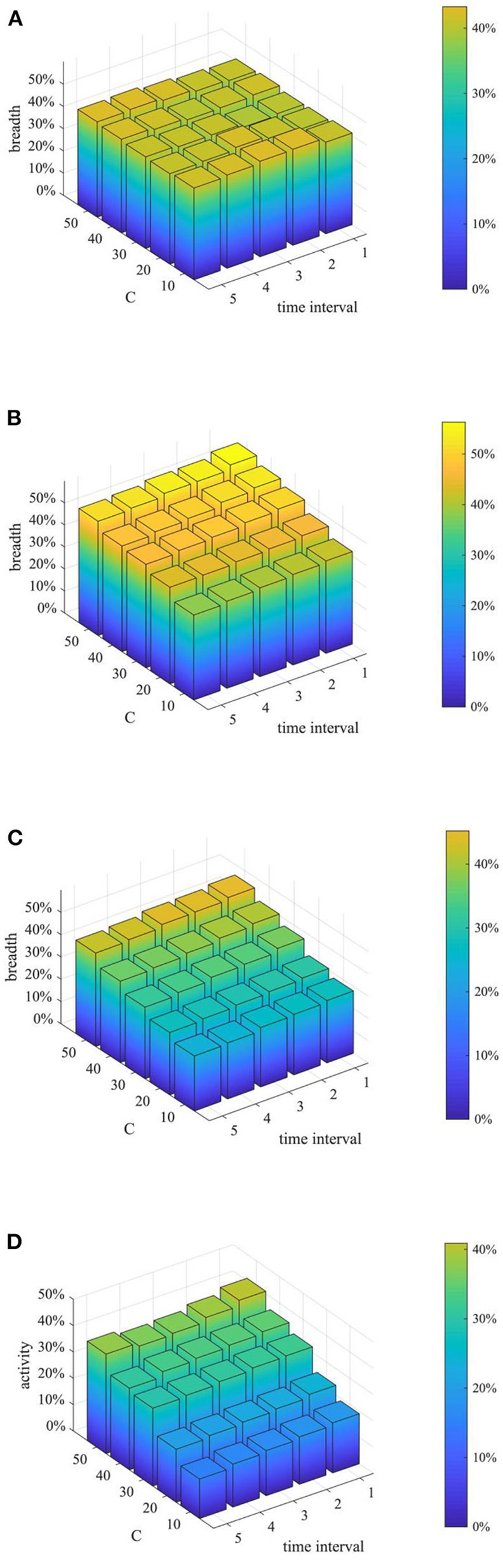
Network activity based on different combination. **(A)** *t* = 10. **(B)** *t* = 20. **(C)** *t* = 30. **(D)** *t* = 40.

Furthermore, considering the urgency and necessity of rumor control, it is necessary to find out the most critical factors in the process of refuting rumors. Based on this, a combined analysis of the number of rumor-refuting individuals and the time interval of rumor-refuting is conducted. Here, the numbers of rumor-refuting individuals are 10, 20, 30, 40, 50, and the time intervals of rumor-refuting are 1, 2, 3, 4, 5, and then combine in pairs to form 25 different strategies. The implementation effects of the combination at *t* = 1, 15, 30, and 45 are compared, and the results are shown in Figures 17–19.

As can be seen from Figures 17–19, when the time interval of rumor-refuting is fixed, as the number of refuting rumor individuals increases, the entire network information content deviation decreases rapidly, the rumor diffusion range is significantly reduced, and the network activity increases significantly. On the other hand, when the number of refuting rumors remains the same as the time changes, the reduction of the time interval of rumor-refuting can speed up the decline of entire network information content deviation and rumor diffusion range, but it has little effect on the final information content deviation and the rumor diffusion range, and it has no obvious effect on the improvement of network activity. This shows that when adopting a rumor-refuting strategy, more attention should be paid to the number of rumor-refuting individuals.

### Analysis and Discussion

In this section, some simulation results and findings are given firstly. Subsequently, the limitations of our study are also discussed.

#### Simulation Results and Findings

Through simulation experiments, the influence of model parameters on the evolution of rumors is analyzed, and the following conclusions are obtained:

(1) The higher average value of the interest correlation between individuals and the event that caused the rumors represents the lower deviation between the network information content and the real information content, and the larger scale of the rumor diffusion range.

(2) Increasing the average network degree of nodes can expand the influence of rumors, but its influence on the rumor diffusion range has a peak.

(3) The higher average trust threshold of all individuals in the network represents the lower entire network information content deviation, and the smaller scale of the rumor diffusion range.

In addition, according to the implementation effects of different rumor control strategies, the following conclusions are obtained:

(1) Before a public opinion incident occurs, adopting a knowledge popularization strategy and a punishment and restriction strategy for the public can effectively minimize the information content deviation and the rumor diffusion range after the public opinion incident occurs. Besides, the rumor control effect of the punishment and restriction strategy is better than that of the knowledge popularization strategy, but its inhibitory effect on network activity is far greater than that of the knowledge popularization strategy.

(2) After a public opinion incident occurs, the government should adopt a strategy of refuting rumors as soon as possible to minimize the impact of rumors. Moreover, when adopting a rumor-refuting strategy, more attention should be paid to the number of rumor-refuting individuals.

#### Limitations

In this study, there are still some shortcomings in simulation analysis as follows: the BA network constructed in the simulation analysis does not consider the growth of nodes in the diffusion of sudden hot events at the initial moment. Therefore, the network structure needs to be further optimized in the follow-up.

## Empirical Analysis

This section selects “Imported Food Safety Issue during the COVID-19 Pandemic” (hereinafter referred to as “Imported Food Safety”) as an example to verify the effectiveness of the SEIR-OM model.

After the outbreak of COVID-19, in order to prevent the import of the virus from abroad, General Administration of Customs People's Republic of China (GACC) has strengthened the testing of imported food. In June 2020, the COVID-19 was detected on the surface of the imported salmon cutting board at the Xinfadi Seafood Wholesale Market in Beijing, which quickly caused panic among Chinese residents, leading to intense discussions on imported food safety issue. Since then, GACC has repeatedly detected positive samples of COVID-19 virus nucleic acid on the outer packaging of imported food or on the surface of the food, which has caused heated discussions on many occasions.

In order to analyze the diffusion of rumors in the “Imported Food Safety” incident, two incidents with a large amount of topic discussion are selected as the analysis objects based on the topic search on the Weibo platform. Firstly, the COVID-19 was detected on the surface of the imported salmon cutting board at the Xinfadi Seafood Wholesale Market in Beijing. Discussions on this incident were mainly focused on June 12, 2020–June 22, 2020. Secondly, the COVID-19 was detected on the surface of the imported cherry in Wuxi, Jiangsu Province in China on January 22, 2021. Discussions on this incident mainly focused on January 22, 2021–February 8, 2021. There are discussions about these two incidents on the Internet, such as “Eating imported food will get COVID-19” and “Eating imported food is dangerous.” In response to these remarks, many Chinese official media continues to quote expert opinions to clarify and refute rumors.

**Figure 20:**
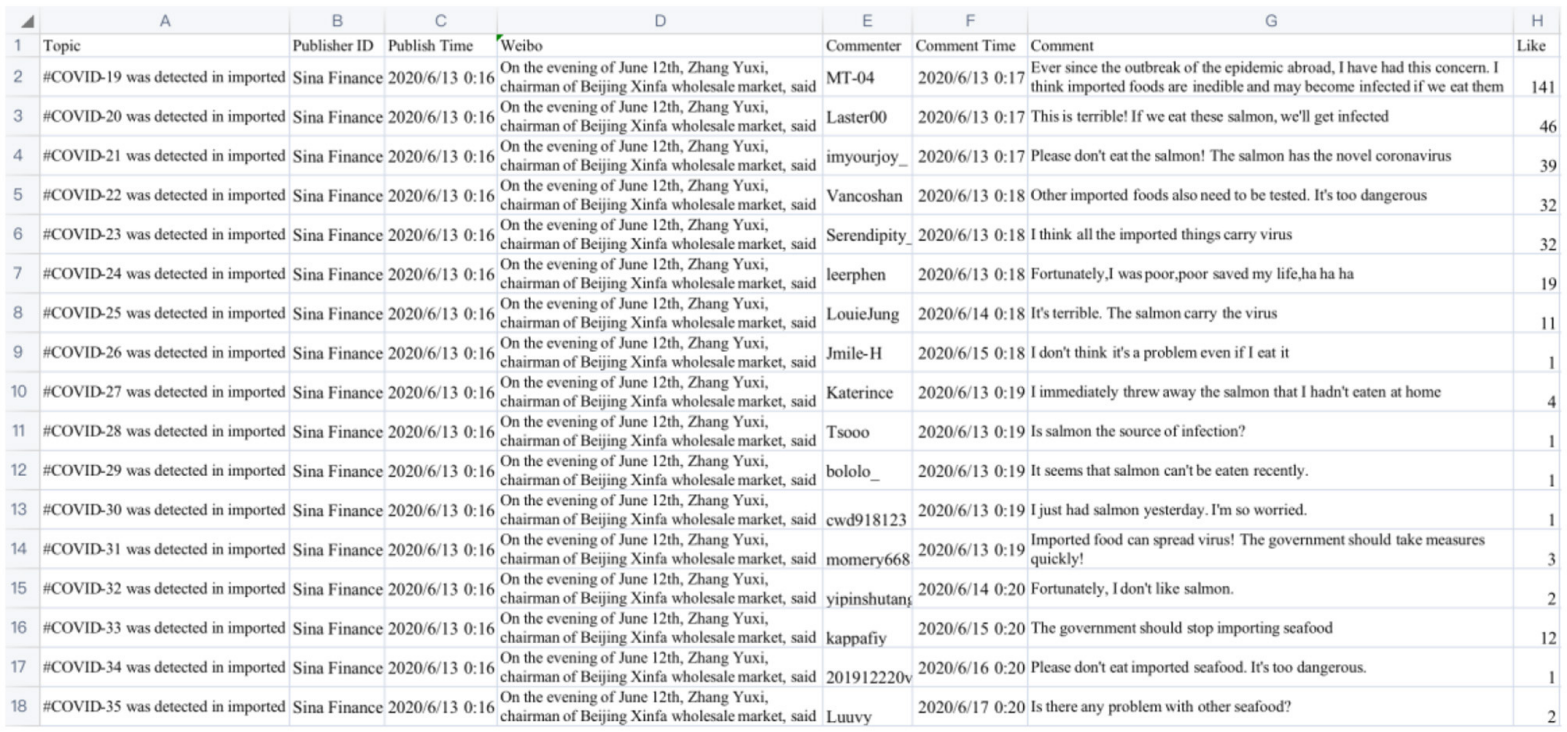
Schematic diagram of the data.

The relevant Weibo data is crawled through python, and a total of 41,351 data is obtained. The schematic diagram of the data is shown in Figure 20.

After obtaining and preprocessing the data, it is necessary to identify the content of the comments. Here, we first establish two corpus sets including rumors and truths related to “Imported Food Safety,” and then use JIEBA (32) word segmentation algorithm and word2vec algorithm to calculate the similarity between the review content and the two corpora sets one by one. If the similarities between the review content and the two corpora sets are low, it will be recognized as an irrelevant comment. If a comment is more similar to the rumor text set than the real content corpus set, it will be recognized as a rumor, otherwise it will be recognized as a truth. After removing irrelevant comments, there are 20,502 pieces of data in the two cases. Although the amount of data here is limited, according to the six-degree separation theory (33) in interpersonal relationships, the statistical results of these user data can reflect the universality of Weibo user behavior to a large extent. The data information involved in the case analysis is shown in Table 4.

**Table 4:** Relevant data.

**Case**	**Time**	**Comments**	**Users**	**Duration**
COVID-19 was detected on the surface of the imported salmon cutting board at the Xinfadi Seafood Wholesale Market in Beijing	From June 12, 2020 to June 22, 2020	8,275	5,601	10 days
COVID-19 was detected on the surface of the imported cherry in Wuxi	From January 22, 2021 to February 8, 2021	12,227	9,134	9 days

In order to verify the validity of the SEIR-OM model constructed in this article, the existing evolutionary game model is introduced and compared with SEIR-OM model. We make the following three assumptions about the evolutionary game model: (1) The individuals in the network are divided into uninformed individuals and informed individuals according to their states. (2) Only the game behavior between informed individuals and uninformed individuals is discussed in the model. (3) There are malicious individuals in the network. The rules of game gains in the model are set as follows: (1) When an informed individual chooses to diffuse information, if the uninformed individual receives the information, the informed individual will get a higher gain *a* (*a* > 1), and the uninformed individual's gain is equal to 1; (2) When an informed individual chooses to diffuse information, if the uninformed individual does not receive the information, the informed individual's gain will be damaged and become −1, and uninformed individual's gain will be 0; (3) When the informed individual does not diffuse information, the gains of both parties are 0; (4) If malicious individuals successfully diffuse rumors to uninformed individuals, they can obtain excess gains; (5) Individuals who diffuse rumors will be punished by the government, and their gains will decrease by *g*. According to the above rules, the gain matrix of the evolutionary game model is shown in Table 5.

**Table 5:** Gain matrix of the evolutionary game model.

				**Uninformed individual**	
				**Receive**	**Not receive**
Informed individual	General individual	Truth	Diffuse	(*a*,1)	(−1,0)
			Not diffuse	(0,0)	(0,0)
		Rumor	Diffuse	(*a-g*,1)	(−1,0)
			Not diffuse	(0,0)	(0,0)
	Malicious individual	Truth	Diffuse	(*a*,1)	(−1,0)
			Not diffuse	(0,0)	(0,0)
		Rumor	diffuse	(*a+d-g*,1)	(−1,0)
			Not diffuse	(0,0)	(0,0)

In addition, the individual strategy update rules in the evolutionary game model are as follows: individual *i* randomly selects a neighbor individual *j*, and imitates the strategy of *j* with a certain probability, as shown in formula (19).


(19) $$\begin{array}{l} W\left(S_{i}\leftarrow S_{j}\right)=\frac{P_{j}-P_{i}}{\max\left(k_{i},k_{j}\right)H} \end{array}$$

where *S*_*i*_ and *S*_*j*_ are the strategies adopted by *i* and *j*; *P*_*i*_ and *P*_*j*_ are the cumulative gains of *i* and *j* after the game; *k*_*i*_, *k*_*j*_ are the degrees of *i* and *j*; *H* is the maximum difference in the game gains between individuals.

Since the evolutionary game model cannot reflect the difference of information content, i.e., it cannot calculate the deviation degree of information content, the comparison content of different models only includes the rumor diffusion range and network activity. In order to make the simulation environment closer to the real situation of the two incidents, some parameters in the two models will be adjusted according to the data of different time periods: (1) According to the Pareto principle (34), 20% of the people in society will produce 80% impact, and malicious individuals play a major role in the rumor diffusion. Therefore, the proportion of malicious individuals in the two models is set to be 20%; (2) The Chinese government takes a strong management measures on online rumors, so the intensity of government punishment *p* is set to 0.5 in the SEIR-OM model, and the government punishment *g* in the evolutionary game model is set to 0.5, too; (3) On June 14, 2020, when the deviation of the online information content of the “COVID-19 was detected on the surface of the imported salmon cutting board in Beijing” reached more than 10%, the official media refuted the rumor for the first time. Therefore, in response to this incident, when the entire network information content deviation is >10%, the government adopts a rumor-refuting strategy. Similarly, for the “the COVID-19 was detected on the surface of the imported cherry in Wuxi, Jiangsu,” it is set that when the entire network information content deviation is >3%, the government adopts a rumor-refuting strategy; (4) As experts keep responding to the doubts about the safety of imported foods, the public's knowledge reserves going up. Therefore, it is assumed that the individual knowledge reserves in the SEIR-OM model obey the Poisson distribution with the mean λ is 1 and 3 in the two time periods, respectively; (5) In these two incidents, the government's time interval for rumor-refuting was 5 and 2 days, respectively. Therefore, the government's rumor-refuting time interval in the SEIR-OM model was set to 5 and 2 days, respectively; (6) Because the difficulty of diffusing rumors after the government has refuted the rumors will increase, the excess gain of malicious individuals diffusing false information will decreases. As a result, the excess gain of malicious individuals diffusing false information in the evolutionary game model before the government refuting the rumors is assumed to be 0.7, and becomes 0.3 after the government refuting the rumors. In addition, the other parameters of the SEIR-OM model are set as: μ_*b*_ = 0.9, μ_*c*_ = 0.5. The other parameters in the evolutionary game model are set as: *a* = 0.12, *H* = 5. It is assumed that the number of simulation network nodes of the two models is both 500.

**Figure 21:**
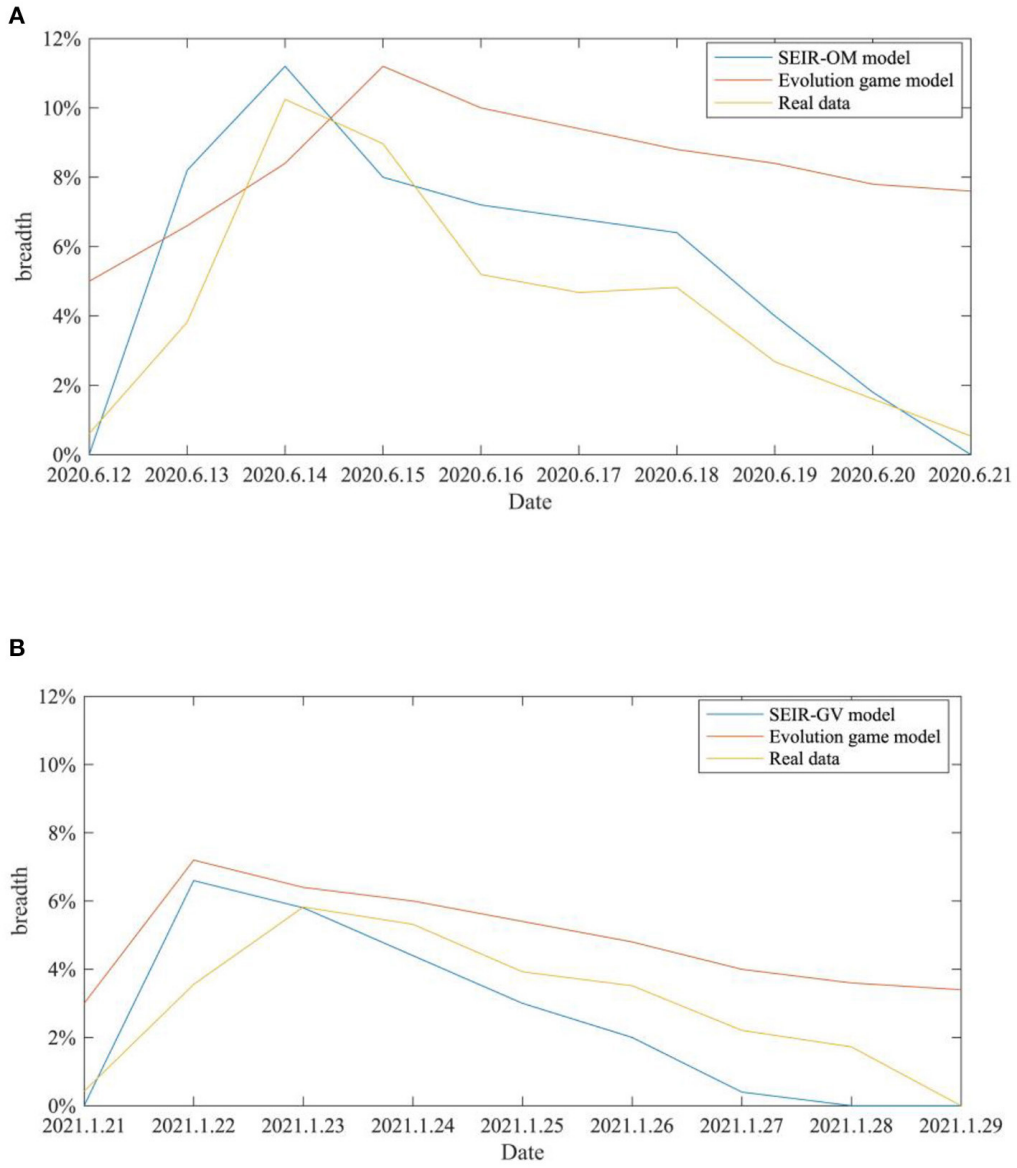
Comparison of the rumor diffusion ranges in two events. **(A)** COVID-19 was detected on the surface of the imported salmon cutting board in Beijing. **(B)** COVID-19 was detected on the surface of the imported cherry in Wuxi.

SEIR-OM model and the evolutionary game model are used to simulate the changes in the rumor diffusion in the two incidents here, and the two change curves are compared with the actual curves shown in Figure 21. In this figure, the blue line represents the rumor diffusion curve simulated by the SEIR-OM model, the red line represents rumor diffusion curve simulated by the evolutionary game model, and the yellow line represents the rumor diffusion curve based on real data. In addition, the Root Mean Square Error (RMSE) is used here to accurately reflect the error between the variation curve of the rumor diffusion range simulated by SEIR-OM model, evolutionary game model and the real data. The results are shown in Table 6.

**Table 6:** RMSE of rumor diffusion scale.

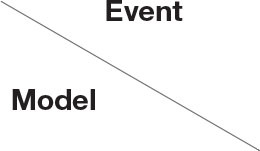	**COVID-19 was detected on the surface of the imported salmon cutting board in Beijing**	**COVID-19 was detected on the surface of the imported cherry in Wuxi**
SEIR-OM model evolutionary game model	0.0186 0.0467	0.0435 0.0683

It can be seen from Figures 21A,B that the rumor diffusion curves simulated by the two models both show an upward trend before the government adopts the intervention strategy, and the curve simulated by the SEIR-OM model rises faster. After the government adopts the intervention strategy, the curve simulated by the evolutionary game model shows a gentle downward trend. In contrast, the curve simulated by the SEIR-OM model declines faster, and the change trend is similar to the real curve. According to Table 6, in terms of rumor diffusion, the error of the simulation results of the SEIR-OM model in the two incidents is smaller than that of the evolutionary game model, and the simulated curve is closer to the real curve, indicating that the SEIR-OM model is closer to real situation in terms of rumor diffusion.

**Figure 22:**
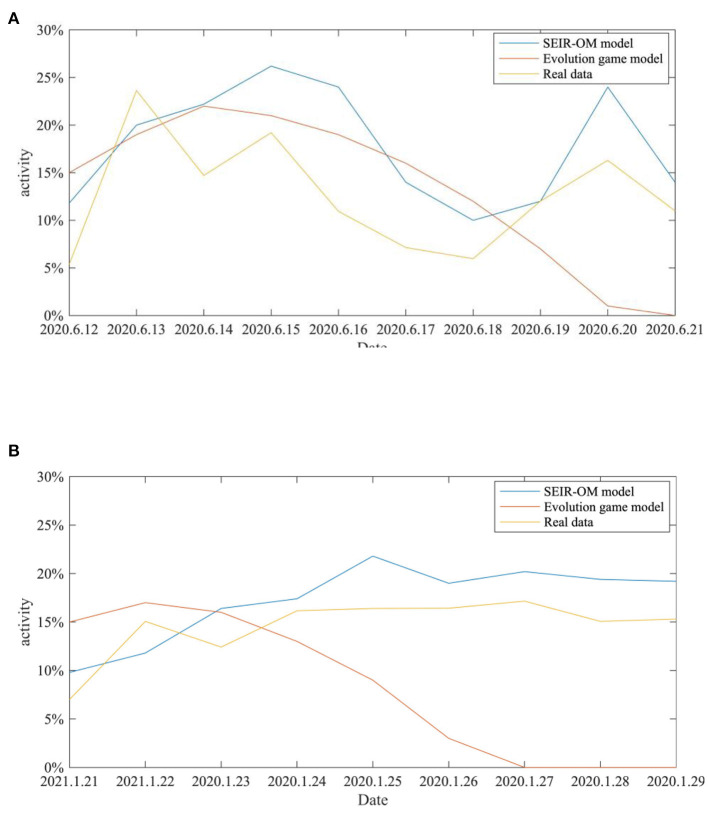
Comparison of the network activities in the two incidents. **(A)** COVID-19 was detected on the surface of the imported salmon cutting board in Beijing. **(B)** COVID-19 was detected on the surface of the imported cherry in Wuxi.

In addition, the SEIR-OM model and the evolutionary game model are used to simulate the changes in network activity in the two events and compare with the real situation shown in Figure 22. In this figure, the blue line represents the network activity curve simulated by the SEIR-OM model, the red line represents the network activity curve simulated by the evolutionary game model, and the yellow line represents the network activity curve drawn based on real data. In addition, the RMSE is used here to accurately reflect the error between the network activity curve simulated by the SEIR-OM model and the evolutionary game model and the real curve. The results are shown in Table 7.

**Table 7:** RMSE of network activity.

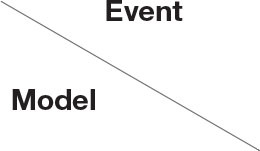	**COVID-19 was detected on the surface of the imported salmon cutting board in Beijing**	**COVID-19 was detected on the surface of the imported cherry in Wuxi**
SEIR-OM model Evolutionary game model	0.068 0.0855	0.0647 0.0832

From Figures 22A,B, it can be seen that the network activity curves of the two events simulated by the evolutionary game model both show an upward trend, and then a downward trend after the government adopts an intervention strategy, and the rate of decline keeps accelerating. In contrast, due to the different frequency of government refuting rumors, the two curves simulated by the SEIR-OM model have certain differences. The simulated network activity curve for the “COVID-19 was detected on the surface of the imported salmon cutting board in Beijing” event has two peaks, while the simulated network activity for the other incident remained stable at about 20% after the government frequently refuted rumors. After comparing the actual curve, it is easy to find that the curve simulated by the SEIR-OM model is closer to the actual curve, indicating that the SEIR-OM model is closer to the real situation in terms of network activity.

**Figure 23:**
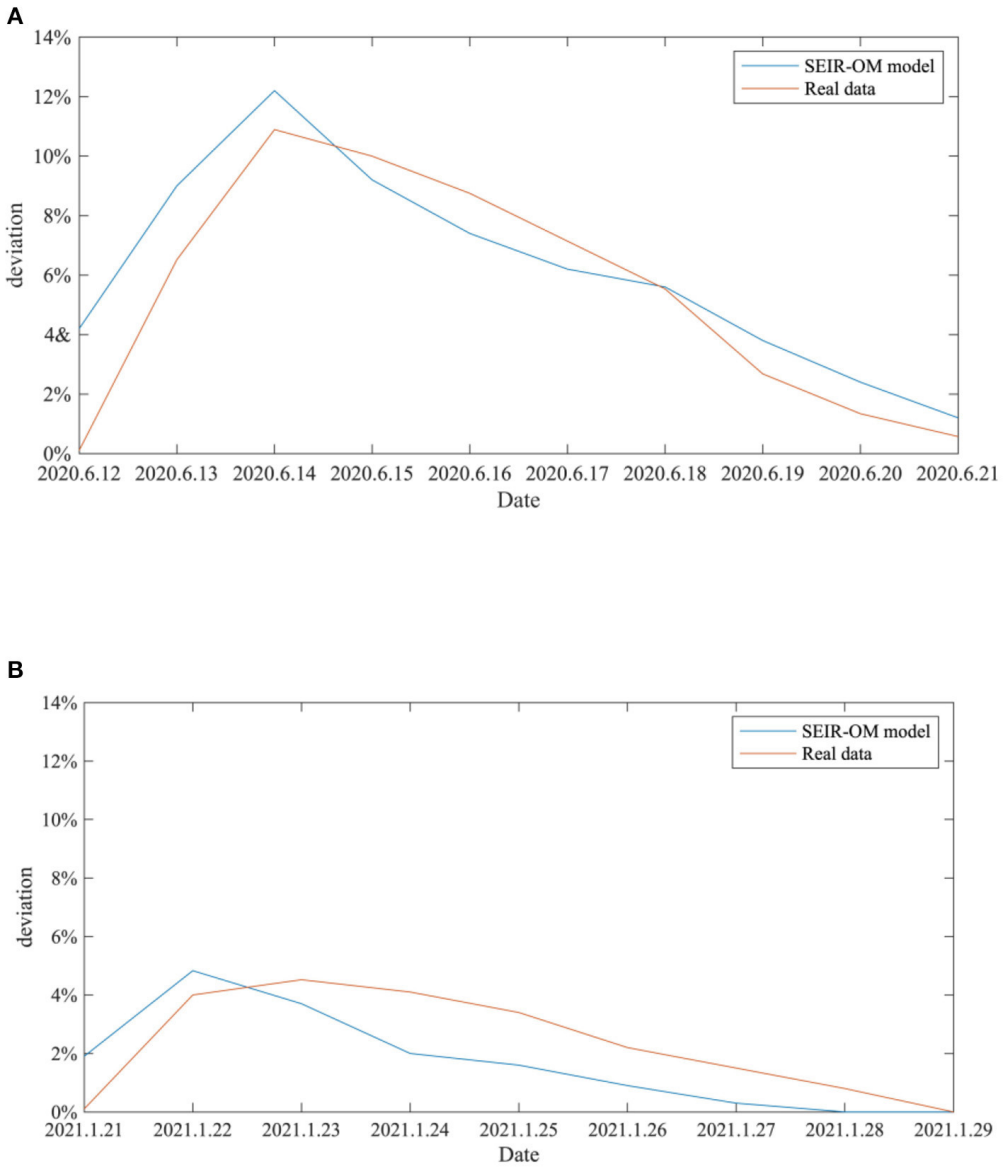
Comparison of entire network information content deviations in two events. **(A)** COVID-19 was detected on the surface of the imported salmon cutting board in Beijing. **(B)** COVID-19 was detected on the surface of the imported cherry in Wuxi.

In addition, in order to reflect the effectiveness of the SEIR-OM model in terms of entire network information content deviation, this curve simulated by the SEIR-OM model is compared with the actual curve, and the result is shown in Figure 23. In Figure 23, the blue line represents the information content deviation curve simulated by the SEIR-OM model, and the red line represents the information content deviation curve drawn based on real data.

It can be seen from Figure 23 that in terms of entire network information content deviation, although the curve simulated by the SEIR-OM model is different from the real data curve, the trend of the two is similar. Therefore, it shows that SEIR-OM model performs well in the entire network information content deviation.

## Conclusions

This article integrates individual heterogeneity factors into the SEIR model, and designs an individual state transition mode at first. Subsequently, based on trust theory and information asymmetry theory, it establishes an individual information interaction mode, and constructs an improved SEIR model named SEIR-OM model. Then the diffusion process of rumors and the implementation effects of different rumor control strategies are simulated and analyzed. Finally, the article verifies the rationality and effectiveness of the SEIR-OM model through the real case from the imported food safety issue during the COVID-19 Pandemic.

However, this article still has the following shortcomings, which need further study:

(1) The BA network constructed in the article only considers the exit of the interconnection among nodes, but does not consider the growth of nodes in the diffusion of sudden hot events at the initial moment. Therefore, the network structure needs to be further optimized in the follow-up.

(2) Rumors in the constructed model are transmitted through random pairwise information interaction between the Internet and the people. In reality, a netizen can send the information to a designated person, or send it in groups to his friends or strangers. Therefore, it is necessary to consider a variety of forms of private information transmission on the Internet, such as group sending, and directional sending.

## Data Availability Statement

The data used to support the findings of this study are available from the corresponding author upon request.

## Author Contributions

TC described the proposed framework and wrote the whole manuscript. JR implemented the simulation experiments. JY collected data. GC revised the manuscript. All authors read and approved the final manuscript.

## Funding

This research was supported by the National Social Science Foundation of China (20BTQ059).

## Conflict of Interest

The authors declare that the research was conducted in the absence of any commercial or financial relationships that could be construed as a potential conflict of interest.

## Publisher's Note

All claims expressed in this article are solely those of the authors and do not necessarily represent those of their affiliated organizations, or those of the publisher, the editors and the reviewers. Any product that may be evaluated in this article, or claim that may be made by its manufacturer, is not guaranteed or endorsed by the publisher.
